# Optimized gene engineering of murine CAR-T cells reveals the beneficial effects of IL-15 coexpression

**DOI:** 10.1084/jem.20192203

**Published:** 2020-11-06

**Authors:** Evripidis Lanitis, Giorgia Rota, Paris Kosti, Catherine Ronet, Aodrenn Spill, Bili Seijo, Pedro Romero, Denarda Dangaj, George Coukos, Melita Irving

**Affiliations:** 1 Department of Oncology, Ludwig Institute for Cancer Research Lausanne, Lausanne University Hospital and University of Lausanne, Lausanne, Switzerland; 2 Department of Oncology, Lausanne University Hospital and University of Lausanne, Lausanne, Switzerland

## Abstract

Limited clinical benefit has been demonstrated for chimeric antigen receptor (CAR) therapy of solid tumors, but coengineering strategies to generate so-called fourth-generation (4G) CAR-T cells are advancing toward overcoming barriers in the tumor microenvironment (TME) for improved responses. In large part due to technical challenges, there are relatively few preclinical CAR therapy studies in immunocompetent, syngeneic tumor-bearing mice. Here, we describe optimized methods for the efficient retroviral transduction and expansion of murine T lymphocytes of a predominantly central memory T cell (T_CM_ cell) phenotype. We present a bicistronic retroviral vector encoding both a tumor vasculature–targeted CAR and murine interleukin-15 (mIL-15), conferring enhanced effector functions, engraftment, tumor control, and TME reprogramming, including NK cell activation and reduced presence of M2 macrophages. The 4G-CAR-T cells coexpressing mIL-15 were further characterized by up-regulation of the antiapoptotic marker Bcl-2 and lower cell-surface expression of the inhibitory receptor PD-1. Overall, this work introduces robust tools for the development and evaluation of 4G-CAR-T cells in immunocompetent mice, an important step toward the acceleration of effective therapies reaching the clinic.

## Teaser

This work presents robust tools for the transduction and expansion of murine CAR-T cells and demonstrates the impact of murine IL-15 co-expression on CAR-T cell expansion, phenotype, function, and tumor microenvironment reprogramming in immunocompetent mice.

## Graphical Abstract

**Figure:**
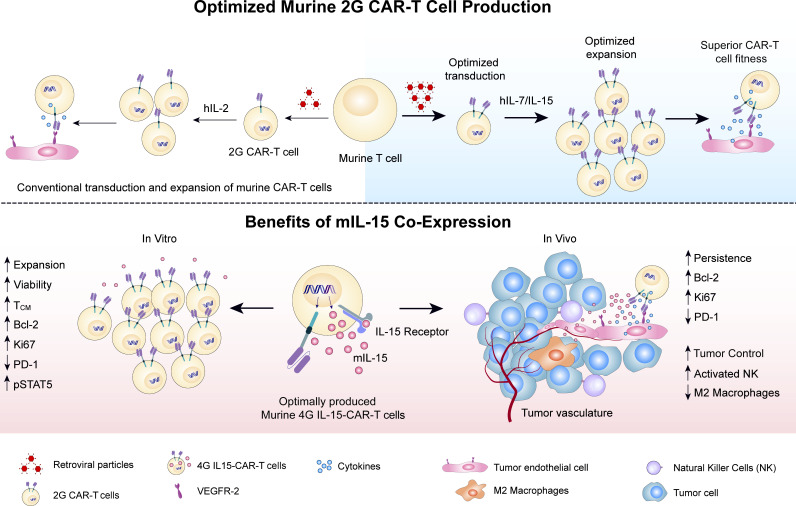

## Introduction

The adoptive cell transfer (ACT) of ex vivo–expanded T lymphocytes has yielded robust and durable clinical responses against several cancer-types, such as tumor-infiltrating lymphocyte therapy of advanced melanoma (Mardiana et al., 2019). Another approach to ACT involves the redirection of peripheral blood T cells to tumor antigens by engineering them to express a chimeric antigen receptor (CAR) that triggers cellular activation upon tumor antigen binding. CAR-T cell therapy against hematologic malignancies, by targeting the B cell lineage antigens CD19 or the B cell maturation antigen, has proven efficacious in the clinic, and there is optimism that similar success will be achieved for some solid tumors (Geyer and Brentjens, 2016; Irving et al., 2017).

A range of physical (Lanitis et al., 2015) and immunometabolic barriers that can prevent T cell homing, transendothelial migration across tumor blood vessels, engraftment/persistence, and effector function limit the potency of CAR-T cell therapy against solid tumors (Brown et al., 2016; Louis et al., 2011). Moreover, chronic antigen exposure and a lack of sufficient costimulation in the tumor microenvironment (TME) can cause CAR-T cell exhaustion (Irving et al., 2017). Coengineering of CAR-T cells may help to overcome some of these obstacles (Lanitis et al., 2020). Genetic modifications, for example, can be made to enable better homing and tumor penetration or render CAR-T cells resistant to suppressive mechanisms in the TME (Stromnes et al., 2010). In addition, CAR-T cells can be armed with secretory molecules or additional receptors to support CAR-T cell activity and/or harness endogenous immunity (Adachi et al., 2018; Pegram et al., 2012).

Preclinical evaluation of CAR-T cells has, for the most part, been performed with xenograft tumor models in immunodeficient mice (Lee et al., 2011; Mardiros et al., 2013; Lanitis et al., 2012). Although this approach can be used to evaluate human CAR-T cell persistence, homing, tumor control, and survival following ACT, critical parameters, including potential toxicity against normal tissues (Tran et al., 2013), and the impact of endogenous immunity on both tumor control and escape are not addressed in such models (Spear et al., 2012; Avanzi et al., 2018). As varying obstacles must be overcome to enhance CAR-T cell responses against different solid tumor types, comprehensive studies in immunocompetent syngeneic tumor models would enable more accurate screening of T cell engineering strategies and provide important insights into improving coengineering and combinatorial treatment approaches (Lanitis et al., 2020).

A key limitation of CAR evaluation in syngeneic models stems from inadequate methodologies for efficient murine T cell transduction and expansion. Indeed, unless T cells derived from multiple donor spleens are transduced or the engineered T cells are restimulated for further expansion, which among other drawbacks are costly and can promote exhaustion and apoptosis (Bucks et al., 2009), respectively, current protocols yield insufficient numbers of CAR-T cells for ACT studies (Lee et al., 2009). The efficiency of cell-surface expression of second-generation (2G) CARs, comprising the endodomain (ED) of CD3ζ and one costimulatory ED (e.g., CD28 or 4-1BB), generally reaches 40–60% (Kochenderfer et al., 2010; Davila et al., 2013; Wang et al., 2014; Fu et al., 2013). Although retroviral transduction rates as high as 70–80% for murine T cells have been reported, this was assessed at 2 to 3 d after transduction (Tran et al., 2013; Kuhn et al., 2019; Kusabuka et al., 2016) and thus may include false positives due to transient expression from nonintegrated vector DNA (i.e., pseudo-transduction; Case et al., 1999, Costello et al., 2000). Moreover, short-term transduction efficiency is often based on reporter genes like GFP, which may overestimate CAR expression levels (Kusabuka et al., 2016; Kuhn et al., 2019; Davila et al., 2013). Finally, while stable retroviral packaging and producer cell lines may enable transduction efficiencies for 2G and third-generation (3G; i.e., a CAR having two or more costimulatory EDs) CARs of >60% (Fu et al., 2013), this is a laborious approach if multiple CAR designs are to be compared (Chinnasamy et al., 2010).

Here, we report the development of an efficient and highly reproducible protocol for primary murine T cell retroviral transduction and expansion, yielding functional murine 2G-CAR-T cells, as well as fourth-generation (4G)-CAR-T cells coengineered to express murine IL-15 (mIL-15) for enhanced in vitro and in vivo function and TME reprogramming. Overall, our work provides important tools for enabling the systematic evaluation of 4G-CAR-T cells in immunocompetent, syngeneic tumor-bearing mice, which we believe is critical for effective therapies reaching the clinic.

## Results

### Optimal retrovirus preparation, T cell activation, and transduction enables efficient cell-surface CAR expression

**Figure 1.**
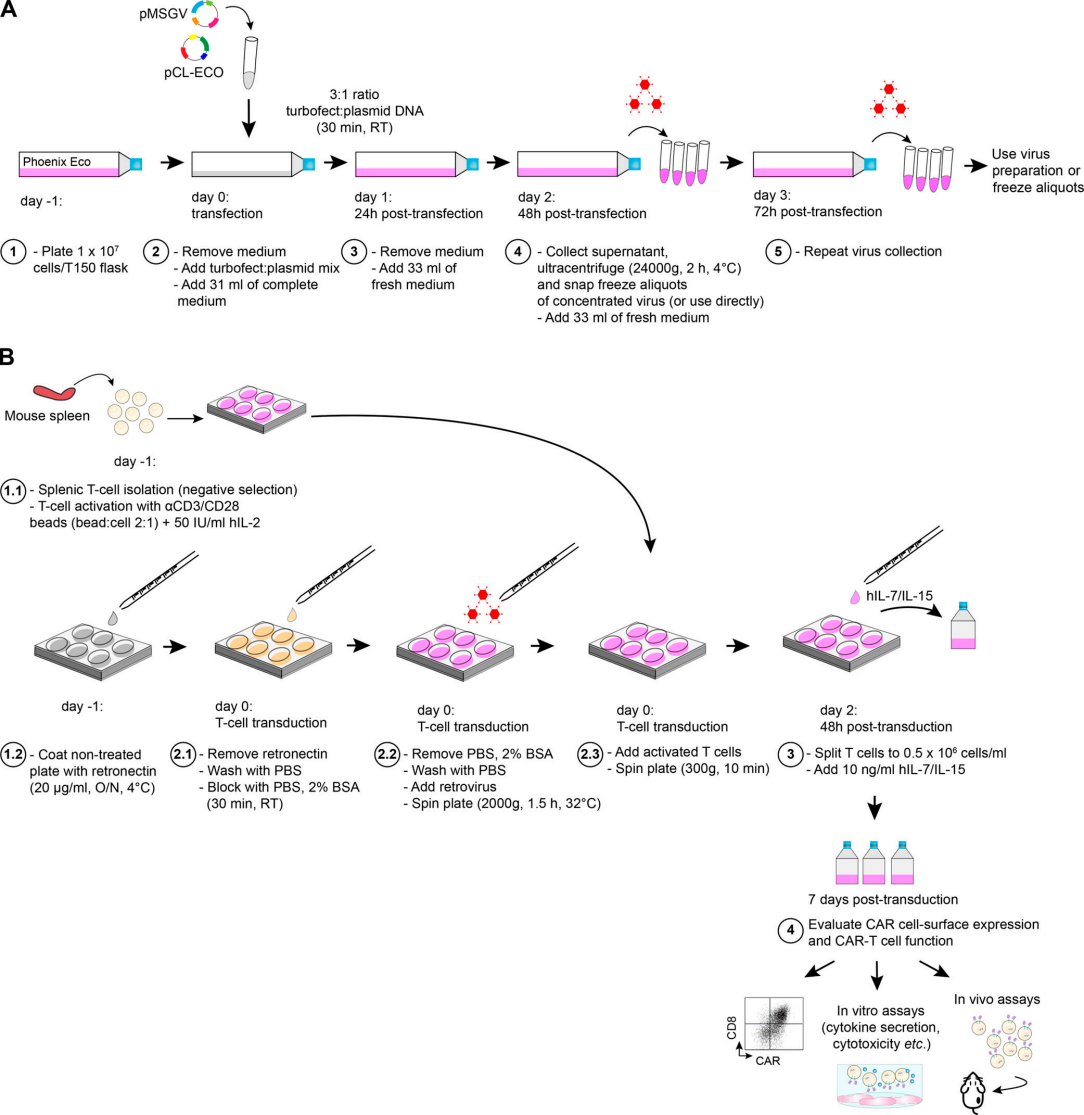
**Overview of retrovirus production and transduction of activated primary murine T cells. (A)** For retrovirus production, plated Phoenix Eco cells are transfected with Turbofect and plasmid mix. After 24 h the medium is refreshed, and at 48 and 72 h, the supernatants are collected, and the virus is concentrated by ultracentrifugation and directly used or frozen. **(B)** For the transduction of primary murine T cells, retrovirus is applied to plates precoated with retronectin and then spun. Activated T cells are then added to the plate, which is subsequently spun. At 48 h after transduction, the T cells are split and hIL-7/IL-15 added to the culture medium. At day 7 after transduction, CAR cell-surface expression is determined by flow cytometry, and the engineered T cells are evaluated in vitro and in vivo. Further details are provided in Materials and methods.

**Figure 2.**
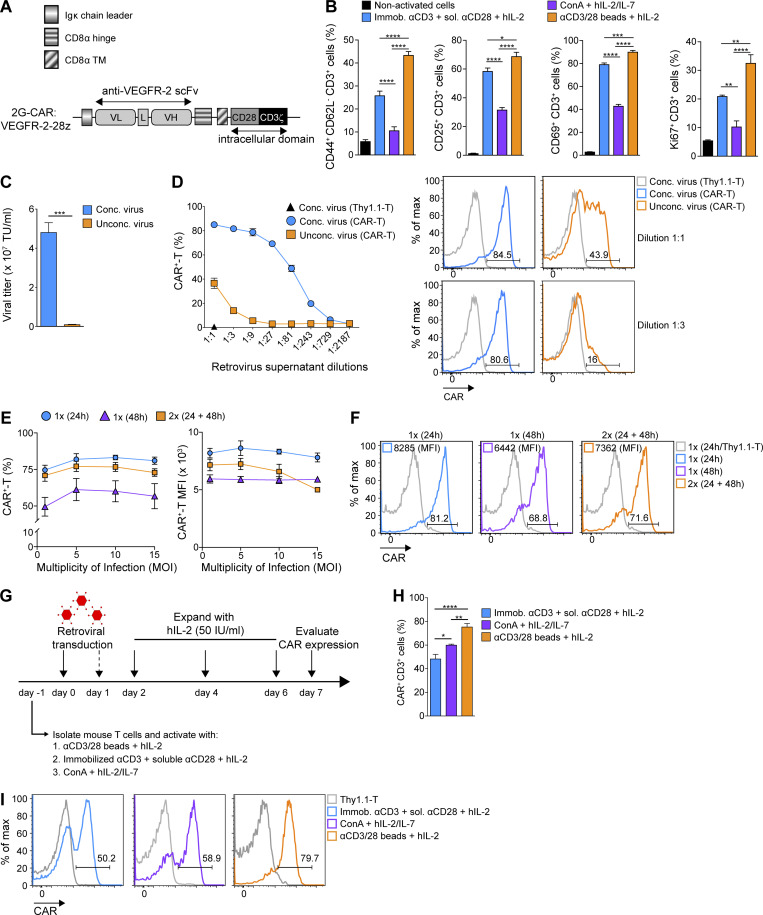
**Concentration of retrovirus and optimal T cell activation increases transduction efficiency. (A)** Diagram of the 2G VEGFR-2–targeted CAR construct (DC101-28z) comprising an Igκ chain leader sequence, the hinge and transmembrane (TM) region of CD8α, the costimulatory ED of CD28, and the ED of CD3ζ (DC101, αVEGFR-2 scFv; L, linker; VH, variable heavy chain; VL, variable light chain). **(B)** Comparison of activation state of differently stimulated T cells. Numbers represent mean percentage of expression ± SEM of T cells from *n* = 6 mice pooled from two independent experiments. **(C)** Virus titer with and without concentration by ultracentrifugation as determined with transduced T cells on day 7 after transduction. Numbers represent mean MOI ± SEM calculated in transduced T cells from *n* = 3 mice. TU, transducing units. **(D)** CAR expression on T cells transduced with serially diluted retrovirus as evaluated by flow cytometric analysis. Numbers represent mean percentage of CAR expression ± SEM of T cells from *n* = 3 mice. Representative histograms of transduced T cells with the indicated dilutions of retroviral supernatants shown. **(E)** Murine T lymphocytes were transduced once (1×) or twice (2×) after activation (24 and/or 48 h) at increasing MOI. Numbers represent mean percentage of MFI CAR ± SEM of T cells from *n* = 3 mice. **(F)** Representative histograms showing CAR frequency and expression level at day 7 after transduction. The experiments in C–F****were repeated three times. **(G)** Schematic diagram of different murine T cell activation and transduction methods tested. **(H)** Evaluation of CAR frequency following different activation methods. Numbers represent mean percentage of CAR ± SEM of T cells from *n* = 5 mice pooled from two independent experiments. **(I)** Representative histograms of CAR expression for differently activated T cells at day 7 after transduction. Statistical analyses were performed using a one-way ANOVA with Tukey post hoc correction test (B and H) and a two-tailed unpaired Student’s *t* test (C): *, P < 0.05; **, P < 0.01; ***, P < 0.001; ****, P < 0.0001.

We sought to optimize murine T cell activation, transduction, and expansion methods for preclinical CAR therapy evaluation in immunocompetent, syngeneic tumor-bearing mice. The final protocol we developed is summarized in Fig. 1 and is described in detail in Materials and methods. We used a 2G-CAR targeting vascular endothelial cell growth factor receptor 2 (VEGFR-2), comprising the well-characterized single-chain variable fragment (scFv) DC101 (Chinnasamy et al., 2010), a CD8α hinge and transmembrane domain, and the murine EDs of CD28 and CD3ζ. The anti-VEGFR-2 CAR retroviral vector is abbreviated as DC101-28z (Fig. 2 A).

**Figure S1.**
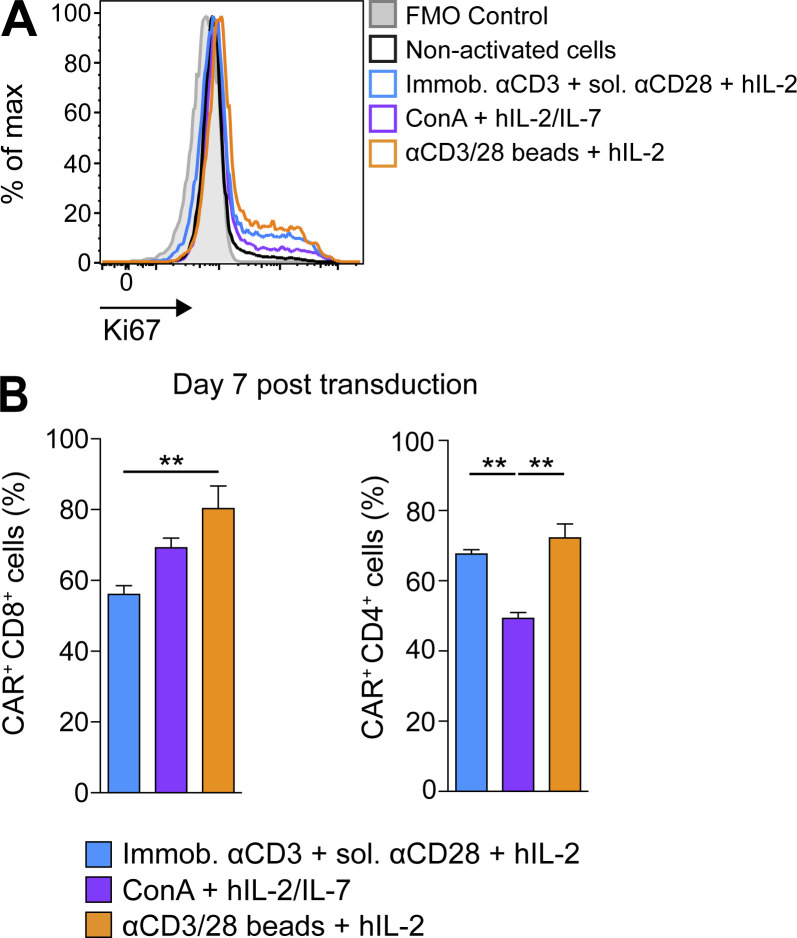
**Stimulation with αCD3/CD28 beads leads to the highest T cell activation and CAR transduction efficiency.** **(A)** Representative histograms of Ki67 expression in differently activated T cells. **(B)** Graph bars present the mean percentage CAR expression ± SEM on differently activated and transduced CD8^+^ and CD4^+^ T cells on day 7 after transduction. All results show mean ± SEM of T cells from *n* = 5 mice (B, left) or *n* = 3 mice (B, right). The experiment was repeated twice. Statistical analyses were performed using a one-way ANOVA with Tukey post hoc correction test: **, P < 0.01. FMO, fluorescence minus one.

Because retroviruses infect proliferating cells (Kusabuka et al., 2016; Chinnasamy et al., 2010; Hu et al., 2017), we first compared three commonly used methods for inducing T cell activation: (i) magnetic beads coated with anti-(α) CD3 antibody (Ab) and αCD28 Ab (αCD3/CD28 beads) plus recombinant human IL-2 (hIL-2), (ii) plate-immobilized αCD3 Ab along with soluble αCD28 Ab (αCD3-plate/CD28) plus hIL-2, and (iii) Concanavalin A plus hIL-2 and hIL-7. Stimulation with αCD3/CD28 beads consistently resulted in the highest frequency of CD44^+^ CD62L^−^ (recently activated, memory), CD25^+^ or CD69^+^ (activated), and Ki67^+^ (proliferating) CD3^+^ T cells (Fig. 2 B and Fig. S1 A).

We next found that concentration of viral particles through ultracentrifugation yielded higher viral titers (>3 × 10^7^ transducing units/ml; Fig. 2 C) and enabled significantly higher transduction of primary activated primary murine T cells as compared with unconcentrated retrovirus (Fig. 2 D), reaching a plateau at a multiplicity of infection (MOI) of 5 (∼80% CAR expression; Fig. 2 E). A single transduction at 24 h after activation versus transduction at both 24 and 48 h did not affect the efficiency in terms of either percentage of cells transduced or CAR expression level per cell (i.e., mean fluorescence intensity [MFI]; Fig. 2, E and F). We observed, however, that the transduction efficiency at 48 h after activation was inferior to that obtained at 24 h after activation (Fig. 2, E and F). A schema of the T cell activation and transduction approaches compared are depicted in Fig. 2 G.

Finally, we observed highest CAR transduction efficiency in CD3^+^ lymphocytes activated with αCD3/CD28 beads in the presence of hIL-2 as compared with the other aforementioned activation methods (Fig. 2, H and I). Similar results were observed for CD8^+^ T cells, while for CD4^+^ T cells, the percentage CAR expression was the same for both αCD3/CD28-bead and αCD3-plate/CD28 activation (Fig. S1 B). Thus, αCD3/CD28-bead activation was used for all further experiments. Notably, we also investigated concentrated lentiviral transduction of αCD3/CD28-bead–activated murine T cells using the same anti-VEGFR-2 CAR, and consistent with another study (Kerkar et al., 2011), we obtained very low transduction efficiency (∼10%, data not shown).

### Culture in the presence of hIL-7 and IL-15 ameliorates murine CAR-T cell expansion, survival, and cytokine production, and promotes a central memory phenotype

**Figure 3.**
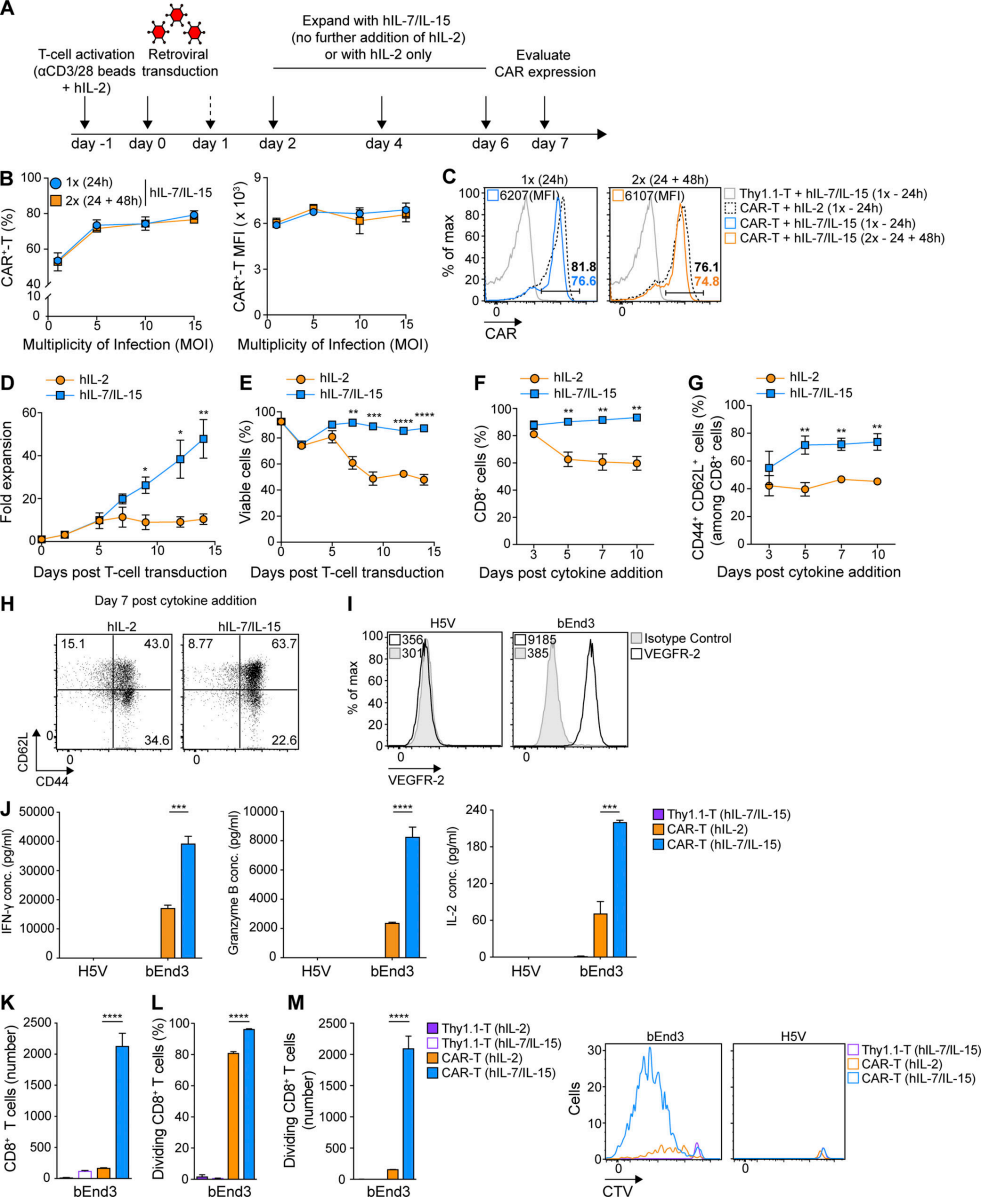
**CAR-T cells cultured with hIL-2 followed by hIL-7/IL-15 exhibit greater expansion, viability, and central memory phenotype than CAR-T cells cultured with hIL-2 only.** **(A)** Overview of primary murine T cell activation, transduction, and expansion. On day 2 after transduction, the culture medium was supplemented with hIL-2 or hIL-7/IL-15. **(B)** CAR expression on splenic murine T cells transduced either at 24 h only, or at 24 and 48 h after activation, at increasing MOI. Values represent mean percentage and MFI CAR expression ± SEM of T cells from *n* = 3 mice. The experiment was repeated twice. **(C)** Representative flow cytometry data of CAR expression on day 7 after the first transduction in transduced T cells cultured in hIL-2 only, or hIL-2 plus hIL-7/IL-15 from day 2 after first transduction. Thy1.1-T cells were used as a negative control. **(D and E)** CAR-T cell expansion (D) and viability (E) upon culture in hIL-2 versus hIL-2 followed by hIL-7/IL-15. The graphs show the mean percentage of expansion (D) or viability (E) ± SEM of T cells from *n* = 4 mice pooled from two independent experiments. **(F)** Percentage of CD8^+^ T cells upon expansion in hIL-2 versus hIL-7/IL-15. Shown are the mean percentage of CD8^+^ T cells ± SEM from *n* = 4 mice. **(G)** Percentage of T_CM_ cells (CD44^+^ CD62L^+^) upon expansion in hIL-2 versus hIL-7/IL-15. Numbers represent the mean percentage of T_CM_ cells ± SEM from *n* = 4 mice pooled from two independent experiments. **(H)** Representative flow cytometric analysis for G on day 7 after cytokine addition. Experiments in D–H were repeated four times. **(I)** Flow cytometric analysis of endothelial cells stained with αVEGFR-2 Ab. The MFI values are indicated. **(J)** Secretion levels of IFN-γ, granzyme B, and IL-2 by CAR-T cells expanded in hIL-2 versus hIL-7/IL-15 upon co-culture with bEnd3 or H5V cells. Graph bars present the mean cytokine concentration (pg/ml) ± SEM from triplicate wells with T cells pooled from *n* = 4 mice. **(K–M)** Persistence (K) and division (L and M) of CTV-stained CAR-T cells expanded in hIL-2 or hIL-7/IL-15 upon co-culture with target cells. Graphs present the mean values ± SEM of T cells from *n* = 3 mice. Representative flow cytometric analysis for the CTV proliferation assay is shown in M. Experiments in I–M were repeated three times. Statistical analyses were performed using a two-tailed unpaired Student’s *t* test (D–G) and a one-way ANOVA with Tukey post hoc correction test (J–M): *, P < 0.05; **, P < 0.01; ***, P < 0.001; ****, P < 0.0001.

While long-term T cell culture in IL-2 drives terminal differentiation, the common γ-chain cytokines IL-7 and IL-15 have been reported to promote a central memory T cell (T_CM_ cell) phenotype enabling superior persistence and in vivo tumor control upon ACT (Klebanoff et al., 2005). Thus, we next compared the expansion and functional properties of transduced murine CAR-T cells cultured in hIL-2 alone versus hIL-2 for the first 3 days, followed by hIL-7/IL-15 for the remainder of the culture period (Fig. 3 A). Both hIL-7 and hIL-15 have been previously demonstrated to act on murine T cells to promote homeostatic proliferation and survival (Eisenman et al., 2002; Nanjappa et al., 2008).

As for hIL-2–expanded CAR-T cells (Fig. 2 G), we observed that a single transduction of T cells at 24 h and subsequent expansion in hIL-7/IL-15 was sufficient to achieve a robust and stable transduction efficiency at a MOI as low as 5 (Fig. 3 B). Both culture conditions (hIL-2 alone versus hIL-2 followed by hIL-7/IL-15) enabled high CAR expression on day 7 (Fig. 3 C). On day 9, however, we observed a 26-fold expansion of CAR-T cells exposed to hIL-7/IL-15 as compared with a 9-fold expansion in the presence of hIL-2 alone at a standard concentration of 50 IU/ml (Fig. 3 D). Moreover, CAR-T cells cultured with hIL-7/IL-15 continued to expand for at least 14 d, while T cells cultured in hIL-2 alone reached a plateau after 1 wk (Fig. 3 D) and exhibited significantly higher levels of cell death starting early in the culture (Fig. 3 E). We also observed a significantly higher frequency of CD8^+^ T cells in the hIL-7/IL-15 culture (Fig. 3 F). Finally, transduced T cells expanded with hIL-7/IL-15 had a significantly higher proportion of T_CM_ cells based on cell-surface expression of the hyaluronic acid receptor CD44 and the L-selectin CD62L from day 5 after cytokine addition (Fig. 3, G and H).

**Figure S2.**
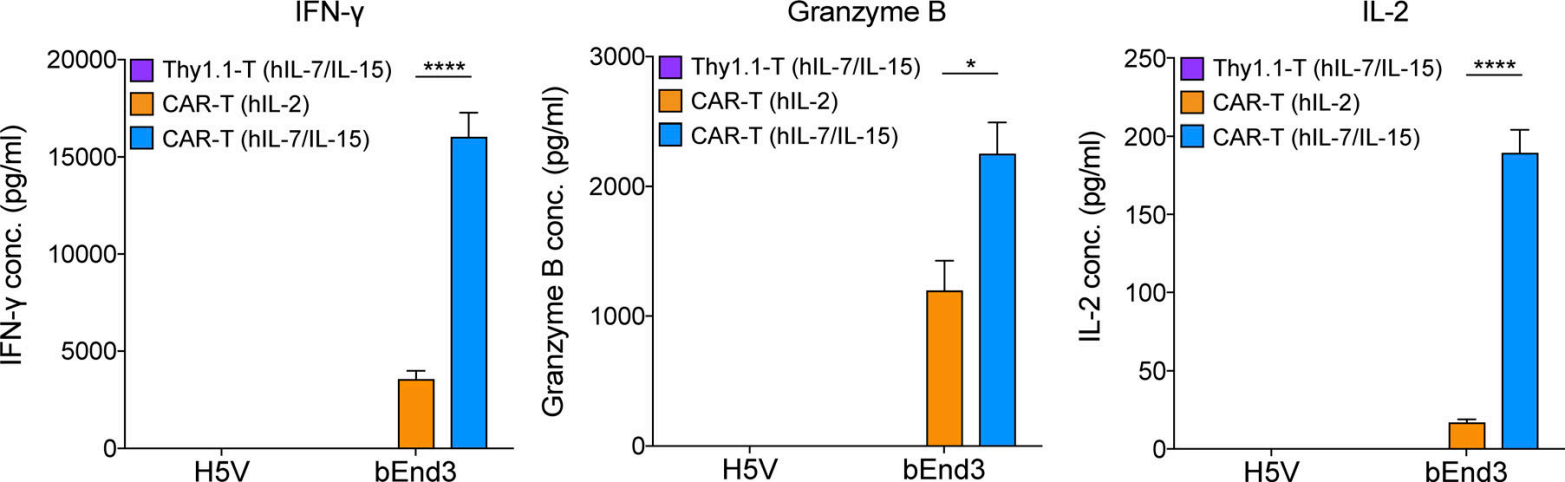
**Enhanced effector functions of CD8^+^ CAR-T cells expanded with hIL-7/IL-15 versus hIL-2.** Secretion levels of IFN-γ, granzyme B, and IL-2 by CD8^+^ CAR-T cells expanded in hIL-2 versus hIL-7/IL-15, upon co-culture with bEnd3 or H5V cells. Graph bars present the mean cytokine concentration (pg/ml) ± SEM of triplicate wells with T cells pooled from *n* = 5 mice. The experiment was repeated twice. Statistical analyses were performed using a one-way ANOVA with Tukey post hoc correction test: *, P < 0.05; ****, P < 0.0001.

We sought to evaluate the in vitro reactivity of hIL-2 only versus hIL-7/IL-15 expanded CAR-T cells against target antigen. On day 7 after transduction, we co-cultured CAR-T cells with bEnd3 murine endothelial cells expressing VEGFR-2, as well as with control VEGFR-2^−^ H5V murine endothelial cells (Fig. 3 I). hIL-7/IL-15 expanded CAR-T cells secreted significantly higher levels of IFN-γ, granzyme B, and IL-2 (Fig. 3 J) after bEnd3 target cell recognition in vitro. Because CAR-T cell expansion with hIL-7/IL-15 results in a higher frequency of CD8^+^ T cells as compared with hIL-2 only, we next sorted CD8^+^ T cells on day 7 after transduction and performed a co-culture with bEnd3 and H5V cells. Higher levels of granzyme B, IL-2, and IFN-γ were secreted by hIL-7/IL-15–expanded CD8^+^ CAR-T cells than hIL-2–expanded ones (Fig. S2). Moreover, hIL-7/IL-15–expanded CAR-T cells exhibited significantly higher persistence (Fig. 3 K), division rates (Fig. 3 L), and numbers of proliferating CD8^+^ T cells after 4 d of co-culture (Fig. 3 M). Thus, as compared with hIL-2 alone, CAR-T cell expansion with hIL-7/IL-15 promotes higher viability and favors a T_CM_ cell phenotype, more robust expansion, and superior secretion of cytokines and long-term proliferative capacity upon challenge with target cells.

### High rate of CAR-T cell transduction is achieved with a 4G-CAR retroviral vector coexpressing mIL-15

The high transduction efficiency achieved with our optimized method encouraged us to evaluate the coexpression of transgenes and test the impact of additional cargo on CAR-T cell performance. Given the enhanced functional properties of CAR-T cells exposed to hIL-7/IL-15 at 48 h after transduction as opposed to hIL-2 alone, we focused on coengineering T cells to constitutively produce mIL-15. Notably, hIL-15 has been previously demonstrated to significantly improve the antitumor activity of human CAR-T cells targeting glioblastoma (Krenciute et al., 2017).

**Figure 4.**
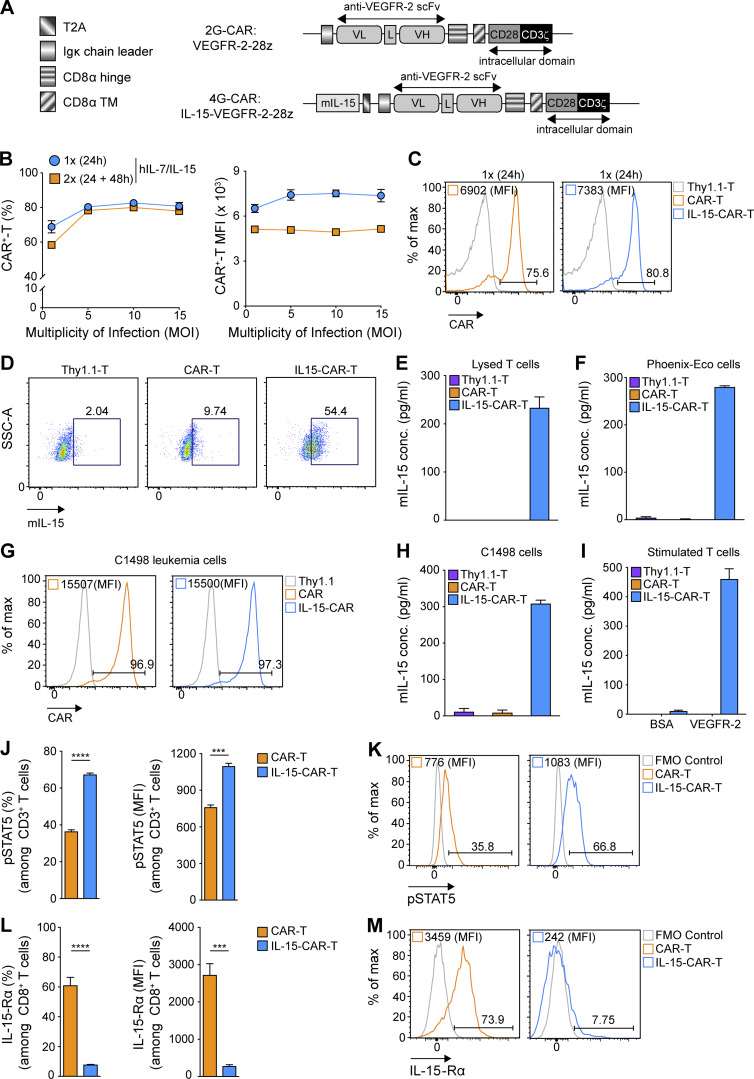
**Murine T cells efficiently transduced to coexpress anti-VEGFR-2 CAR and functionally active mIL-15. (A)** Comparison of the 2G-CAR construct (DC101-28z) and a bicistronic vector encoding both mIL-15 and the anti-VEGFR-2 CAR (4G-CAR construct). **(B)** Murine T cells were transduced once (1×) or twice (2×) with 4G-CAR retrovirus at 24 h or 24 and 48 h after activation at increasing MOI. Numbers represent mean percentage and MFI of CAR ± SEM for T cells from *n* = 3 mice. The experiment was repeated twice. **(C)** Representative flow cytometry data of 2G- and 4G-CAR expression by murine T cells on day 7 after transduction. **(D)** Representative flow cytometry plots showing intracellular mIL-15 production by 4G-CAR-T cells as compared with 2G-CAR-T cells and Thy1.1-T cells. The experiment was performed using T cells pooled from *n* = 4 mice and was repeated three times. **(E)** mIL-15 protein expression by 4G-CAR-T cells determined by ELISA of the supernatant of lysed T cells. Results are presented as the mean concentration (pg/ml) ± SEM from triplicate wells with T cells pooled from *n* = 4 mice. The experiment was repeated twice. **(F)** Evaluation of mIL-15 levels in the supernatant of Phoenix cells 72 h after transfection. Results are presented as the mean concentration (pg/ml) ± SEM of triplicate wells. The experiment was repeated four times. **(G)** Flow cytometry histograms showing CAR expression on C1498 murine leukemia cells transduced to express 2G- or 4G-CAR or Thy1.1. **(H)** mIL-15 detected in the supernatant of 4G-CAR engineered C1498 leukemia cells. Results show the mean concentration (pg/ml) ± SEM of triplicate wells. **(I)** Measurement of mIL-15 secretion by 2G- and 4G-CAR-T cells at 24 h after stimulation with recombinant mVEGFR-2 or BSA. Numbers represent mean concentration (pg/ml) ± SEM of triplicate wells with T cells pooled from *n* = 5 mice. **(J)** Phosphorylated (p) STAT5 expression by 2G- versus 4G-CAR-T cells on day 2 after transduction. Numbers represent mean percentage and MFI pSTAT5 ± SEM of T cells from *n* = 3 mice. **(K)** Representative flow cytometry histograms showing pSTAT5 frequency and levels in 2G- or 4G-CAR-T cells. **(L)** IL-15-Rα expression by 2G- versus 4G-CAR-T cells on day 5 after transduction in the absence of exogenous cytokines. Numbers represent mean frequency and MFI of IL-15-Rα ± SEM of T cells from *n* = 4 mice. **(M)** Representative flow cytometry histograms showing IL-15-Rα frequency and levels for 2G- or 4G-CAR-T cells. Experiments in G–M were repeated three times. Statistical analyses in J and L were performed using a two-tailed unpaired Student’s *t* test: ***, P < 0.001; ****, P < 0.0001. FMO, fluorescence minus one; SSC-A, side scatter area.

**Figure S3.**
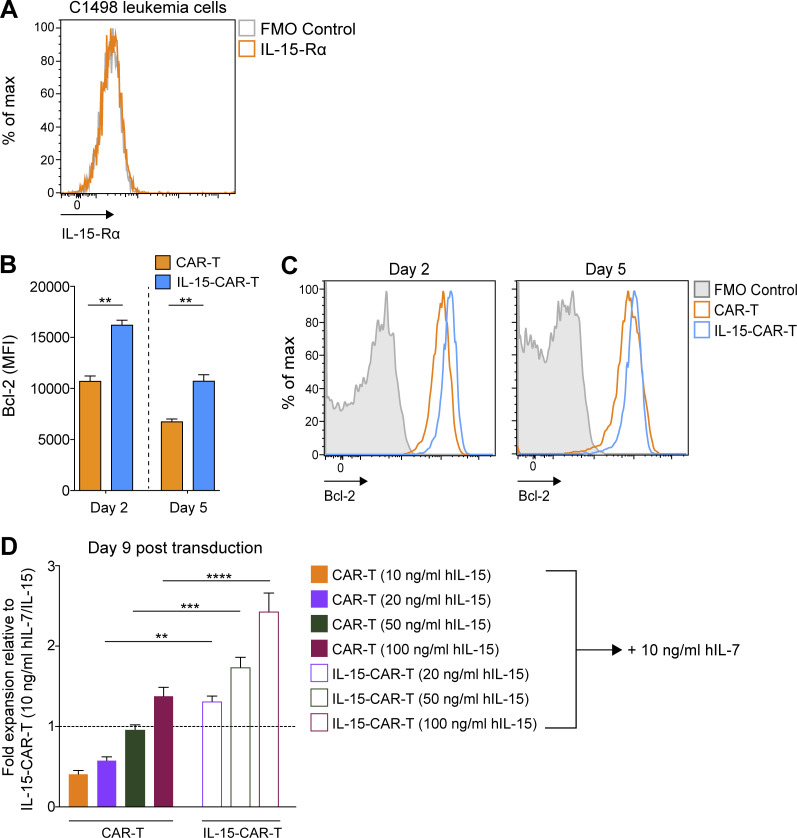
**Enhanced properties of 4G-CAR-T cells efficiently coexpressing mIL-15. (A)** Assessment of IL-15-Rα expression on murine C1498 leukemia cells. Representative flow cytometry histogram of IL-15-Rα expression on C1498 cells. **(B)** Graph bars present the mean MFI of Bcl-2 ± SEM in 2G- versus 4G-CAR-T cells on days 2 and 5 after transduction from *n* = 3 mice. **(C)** Representative histograms showing intracellular levels of Bcl-2 in CD8^+^ 2G- versus 4G-CAR-T cells. The experiments in A–C were repeated three times. **(D)** Fold expansion of 2G- and 4G-CAR-T cells (by day 9 after transduction) cultured in the indicated concentrations of hIL-15 (10, 20, 50, and 100 ng/ml) and 10 ng/ml hIL-7 relative to 4G-CAR-T cells expanded in medium supplemented with 10 ng/ml hIL-7/IL-15. Shown are the mean values of relative fold expansion ± SEM of T cells from *n* = 5 mice. The experiment was repeated twice. Statistical analyses were performed using a two-tailed unpaired Student’s *t* test (B) and a one-way ANOVA with Tukey post hoc correction test (D): **, P < 0.01; ***, P < 0.001; ****, P < 0.0001. FMO, fluorescence minus one.

A bicistronic retroviral vector encoding mIL-15 and the DC101 CAR, both driven by the 5′ LTR of the retrovirus (de Felipe et al., 1999) and separated by a self-cleaving 2A peptide sequence (T2A; Liu et al., 2017), was built to express this 4G-CAR construct (Fig. 4 A). With a single round of transduction at a MOI as low as 5, we achieved a similarly high expression of the 4G- as the 2G-CAR (Fig. 4, B and C), as well as high intracellular expression of mIL-15 (Fig. 4 D). Significant mIL-15 was also detected by ELISA upon lysis of 4G-CAR-T cells (Fig. 4 E), but very low levels of mIL-15 were found in the culture supernatant (data not shown), presumably due to sequestration of the cytokine by cell-surface IL-15 receptor-α (IL-15-Rα), as has been previously observed for human T cells engineered to secrete hIL-15 (Markley and Sadelain, 2010). Our hypothesis was supported by the fact that we detected high levels of soluble mIL-15 in the supernatants of transfected human Phoenix Eco cells (i.e., the retrovirus producer cell line; Fig. 4 F). Moreover, 4G-CAR–transduced C1498 leukemia cells (which do not express IL-15-Rα; Fig. S3 A) secreted high levels of mIL-15 (Fig. 4, G and H). Finally, we activated both 2G- and 4G-CAR-T cells with cognate antigen and found significant secretion of mIL-15 by the 4G-CAR-T cells (Fig. 4 I), as has similarly been reported in the context of engineered human T cells (Krenciute et al., 2017).

### Coexpression of mIL-15 confers enhanced CAR-T cell fitness

**Figure 5.**
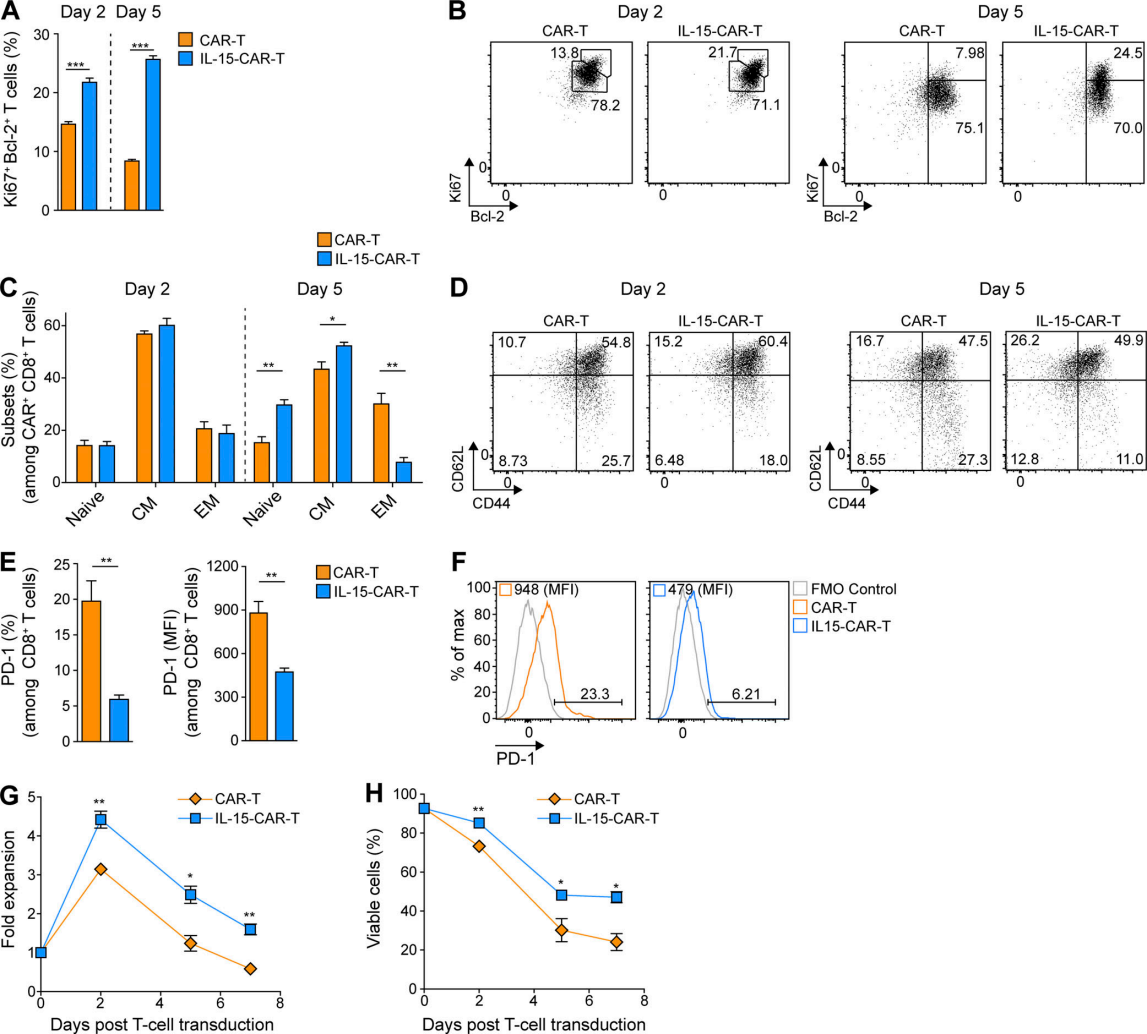
**4G-CAR-T cells coexpressing mIL-15 exhibit enhanced in vitro fitness as compared with 2G-CAR-T cells. (A)** Ki67 and Bcl-2 expression by 2G- versus 4G-CAR-T cells on days 2 and 5 after transduction in the absence of exogenous cytokines. Results presented are mean percentage ± SEM of T cells from *n* = 3 mice. **(B)** Representative flow cytometric analyses of 2G- and 4G-CAR-T cells stained with αKi67 and αBcl-2 Abs on days 2 and 5 after transduction. **(C)** Percentages of naive (CD44^low^ CD62L^high^), CM (CD44^high^ CD62L^high^), and EM (CD44^high^ CD62L^low^) of 2G- versus 4G-CAR-T cells on days 2 and 5 after transduction in the absence of exogenous cytokines. The analysis was performed using T cells derived from *n* = 3 mice. **(D)** Representative flow cytometry plots showing CD44 and CD62L expression on 2G- and 4G-CAR-T cells. Experiments in A–D****were repeated three times. **(E)** PD-1 expression by 2G- versus 4G-CAR-T cells on day 5 after transduction in the absence of exogenous cytokines. Results present the mean percentage and MFI of PD-1 ± SEM of T cells from *n* = 3 mice. **(F)** Representative flow cytometry histograms showing PD-1 frequency and MFI for 2G- versus 4G-CAR-T cells. **(G and H)** Fold expansion (G) and viability (H) of 2G- versus 4G-CAR-T cells in the absence of exogenous cytokines. Graphs present the mean fold expansion (G) or percentage of viability (H) ± SEM of T cells from *n* = 3 mice. Experiments in E–H were repeated four times. Statistical analyses in A, C, E, G, and H were performed using a two-tailed unpaired Student’s *t* test: *, P < 0.05; **, P < 0.01; ***, P < 0.001.

We next sought to investigate the impact of mIL-15 coexpression on CAR-T cell signaling and phenotype. In the absence of exogenous cytokine in the culture supernatant, we observed elevated pSTAT5 in the 4G- versus 2G-CAR-T cells both in terms of frequency and level per cell (Fig. 4, J and K). We further evaluated IL-15-Rα expression and detected lower levels on 4G-CAR-T cells (Fig. 4, L and M), presumably due to receptor internalization (Dubois et al., 2002) and/or mIL-15 occupancy blocking the Ab binding site. Subsequently, we assessed expression of the antiapoptotic protein Bcl-2, previously reported to enhance 2G- versus first-generation (1G)–CAR-T cell persistence (Song et al., 2012), and found higher expression levels on days 2 and 5 after transduction for 4G- as compared with 2G-CAR-T cells in the absence of exogenous cytokines (Fig. S3, B and C). In addition, we observed significantly higher frequencies of Ki67^+^ Bcl-2^+^ 4G-CAR-T cells on days 2 and 5 after transduction (Fig. 5, A and B). Thus, mIL-15 coexpression appears to augment both CAR-T cell survival and proliferation.

We further assessed the phenotype of CAR-T cells in the absence of exogenous cytokines in the culture medium and found that on day 2 following transduction, 2G- and 4G-CAR-T cells displayed no differences in the proportion of naive (CD62L^high^ CD44^low^), central memory (CM; CD62L^high^ CD44^high^) and effector memory (EM; CD62L^low^ CD44^high^) T cell phenotype populations. However, by day 5 after transduction, 4G-CAR-T cells had a higher proportion of naive and CM cells and fewer EM cells, as compared with 2G-CAR-T cells (Fig. 5, C and D). Notably, there were significantly lower levels of the inhibitory receptor programmed cell death 1 (PD-1; both percentage and MFI) on 4G- compared with 2G-CAR-T cells (Fig. 5, E and F).

Consistent with the above findings, we observed that in the absence of exogenous cytokine the 4G-CAR-T cells exhibited increased expansion during the first 2 d after transduction as compared with the 2G-CAR-T cells (Fig. 5 G). Both 2G- and 4G-CAR-T cells began to contract at a similar rate from day 2 after transduction, but there were significantly more 4G- than 2G-CAR-T cells on days 5 and 7 (Fig. 5 G). Finally, we observed higher viability of 4G-CAR-T cells over time (Fig. 5 H). Thus, with our optimized protocol, we achieved a high rate of T cell transduction with retrovirus coexpressing a CAR and mIL-15, and in the absence of exogenous cytokines, these 4G-CAR-T cells exhibit a less differentiated and inhibitory phenotype as well as enhanced expansion and viability in vitro.

### Coexpression of mIL-15 by CAR-T cells boosts expansion and enhances in vitro cytokine production and proliferation

**Figure 6.**
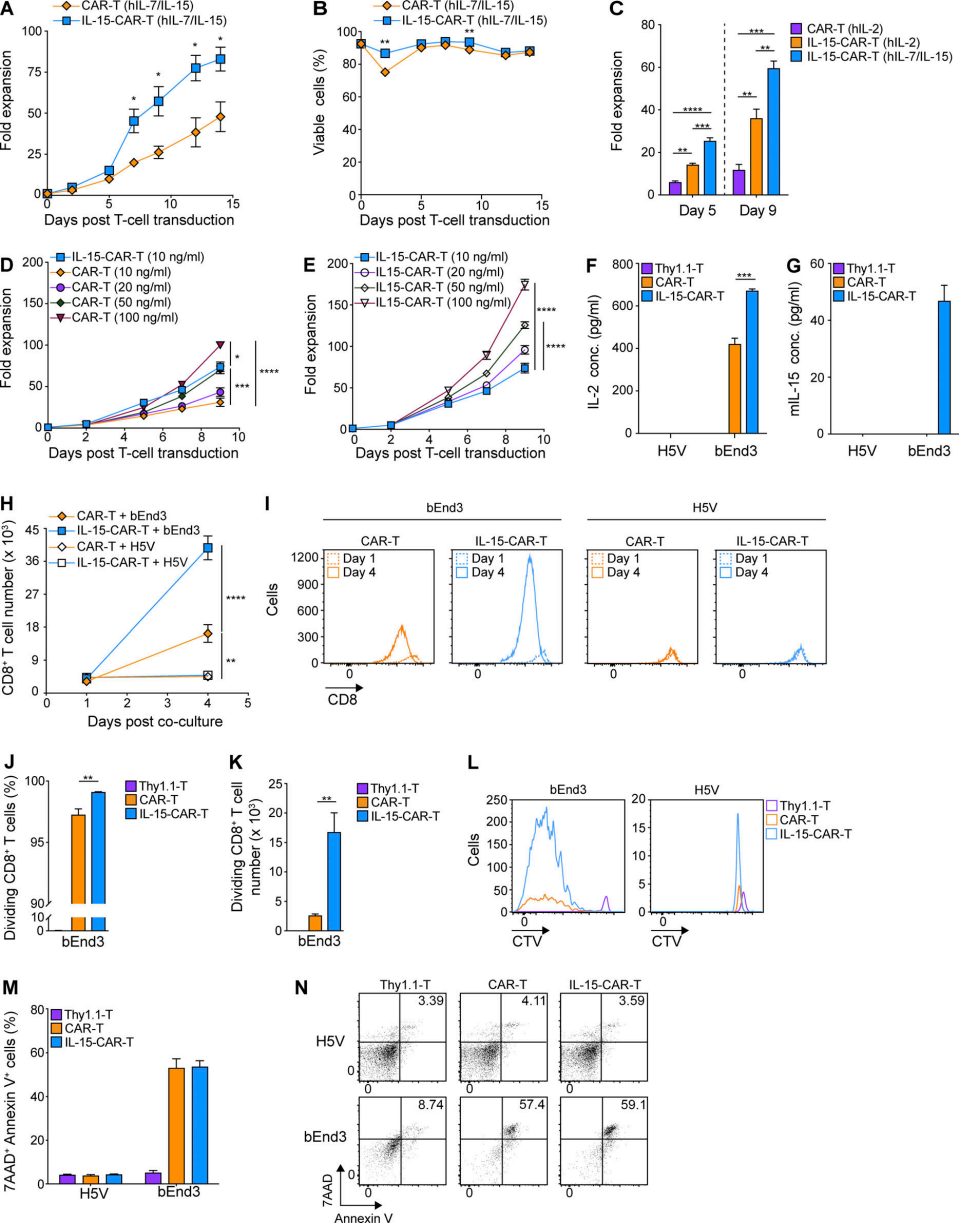
**4G-CAR-T cells coexpressing mIL-15 exhibit robust expansion during culture and enhanced IL-2 secretion and proliferation in response to antigenic stimulation.** **(A and B)** Expansion (A) and viability (B) of 2G- versus 4G-CAR-T cells in the presence of exogenous hIL-7/IL-15 from day 2 after transduction. Results present the mean fold expansion (A) or percentage of viable cells (B) ± SEM of T cells from *n* = 4 mice pooled from two independent experiments. Experiments in A and B were repeated four times. **(C)** Expansion of 2G- versus 4G-CAR-T cells in the presence of the indicated cytokines. Graphs present the mean fold expansion ± SEM of T cells from *n* = 3 mice on days 5 and 9 after transduction. The experiment was repeated three times. **(D)** Expansion of 2G-CAR-T cells in T cell medium supplemented with the indicated concentrations of hIL-15 (10, 20, 50, and 100 ng/ml) and 10 ng/ml hIL-7 in comparison to 4G-CAR-T cells expanded with 10 ng/ml hIL-7/IL-15. Cytokines were added from day 2 after transduction and replenished every 2–3 d. Shown are the mean fold expansion values ± SEM of T cells from *n* = 5 mice. **(E)** Expansion of 4G-CAR-T cells cultured in the indicated concentrations of hIL-15 and 10 ng/ml hIL-7. Shown are the mean values of fold expansion ± SEM of T cells from *n* = 5 mice. Experiments in D and E were repeated twice. **(F)** IL-2 production by 2G- versus 4G-CAR-T cells upon co-culture with target cells. Results present the mean concentration (pg/ml) ± SEM in triplicate wells with T cells pooled from *n* = 4 mice. **(G)** Measurement of mIL-15 secretion by 2G- and 4G-CAR-T cells at 24 h after co-culture with target cells at a CAR^+^ T cell to target cell ratio of 2:1. Numbers represent mean concentration (pg/ml) ± SEM in co-culture supernatants with T cells from *n* = 4 mice. **(H)** Expansion of 2G- versus 4G-CAR-T cells after co-culture with target cells. Results present the mean number of CD8^+^ T cells ± SEM for *n* = 5 mice. **(I)** Shown are representative flow cytometric examples indicating the numbers of CD8^+^ T cells in the co-cultures of 2G- and 4G-CAR-T cells with target cells on days 1 and 4 after co-culture. **(J and K)** Percentage (J) or number (K) of dividing CD8^+^ T cells upon co-culture with bEnd3 cells. Results shown are mean values ± SEM of T cells from *n* = 4 mice. **(L)** Shown is a representative flow cytometric analysis of CTV-stained CAR-T cells co-cultured with bEnd3 or H5V cells. **(M)** Quantification of target cell apoptosis on day 3 after co-culture with 2G- or 4G-CAR-T cells using Annexin V and 7-AAD staining. The graph presents mean values ± SEM of Annexin V^+^/7-AAD^+^ target cells upon co-culture with T cells from *n* = 3 mice. **(N)** Representative dot plots of Annexin V^+^/7-AAD^+^ target cells following CAR-T cell co-culture. Experiments in F–N were repeated three times. Statistical analyses were performed using a two-tailed unpaired Student’s *t* test (A and B) and a one-way ANOVA with Tukey post hoc correction test (C–F, H, J, and K): *, P < 0.05; **, P < 0.01; ***, P < 0.001; ****, P < 0.0001.

We next sought to evaluate the expansion of 4G- versus 2G-CAR-T cells in the presence of exogenous hIL-7/IL-15. We observed continuous expansion of 4G- and 2G-CAR-T cells for 2 wk but at a significantly higher rate for the 4G-CAR-T cells (Fig. 6 A). Viability was similarly high for both over a 10-d period (Fig. 6 B). Notably, 4G-CAR-T cells cultured in hIL-2 demonstrated enhanced expansion at days 5 and 9 as compared with similarly cultured 2G-CAR-T cells (Fig. 6 C). We subsequently sought to determine if increasing hIL-15 levels in the medium could augment 2G-CAR-T cell expansion. We demonstrated that 2G-CAR-T cells cultured in the presence of increasing concentrations of hIL-15 (while maintaining hIL-7 at 10 ng/ml) achieved significant increases in fold expansion, reaching or surpassing that of 4G-CAR-T cells (cultured in standard 10 ng/ml hIL-15) at day 9 after transduction in the presence of 50 ng/ml or 100 ng/ml hIL-15, respectively (Fig. 6 D and Fig. S3 D). Notably, increasing the concentration of hIL-15 in the culture medium from 10 to 50 or 100 ng/ml significantly increased the expansion of 4G-CAR-T cells (Fig. 6 E), and the fold expansion of 4G-CAR-T cells was nearly double compared to that of 2G-CAR-T cells (cultured in equivalent increased hIL-15 concentrations) on day 9 after transduction (Fig. 6 E and Fig. S3 D).

We next tested the effector capacity of 4G- as compared with 2G-CAR-T cells against target cells. Significantly higher levels of IL-2 were produced by 4G- than 2G-CAR-T cells upon co-culture with VEGFR-2^+^ bEnd3 cells at 1 wk after transduction, while neither reacted against VEGFR-2^−^ H5V cells (Fig. 6 F). We further observed mIL-15 secretion by 4G-CAR-T cells only upon co-culture with bEnd3 cells and not H5V cells (Fig. 6 G). In addition, there was significantly higher expansion of 4G- than 2G-CAR-T cells at day 4 after co-culture with bEnd3 cells, and neither expanded upon co-culture with H5V cells (Fig. 6, H and I). The 4G-CAR-T cells also exhibited significantly higher proliferation (Fig. 6 J) and numbers of dividing CD8^+^ T cells compared with 2G-CAR- or control T cells at day 4 of the co-culture (Fig. 6, K and L). The ability of 4G- and 2G-CAR-T cells to induce apoptosis of target cells was equivalent (Fig. 6 M, and N), in accordance with previous evaluation of hIL-15-CAR-T cells (Krenciute et al., 2017).

### CAR-T cells coexpressing mIL-15 exhibit increased in vivo persistence and tumor control

**Figure 7.**
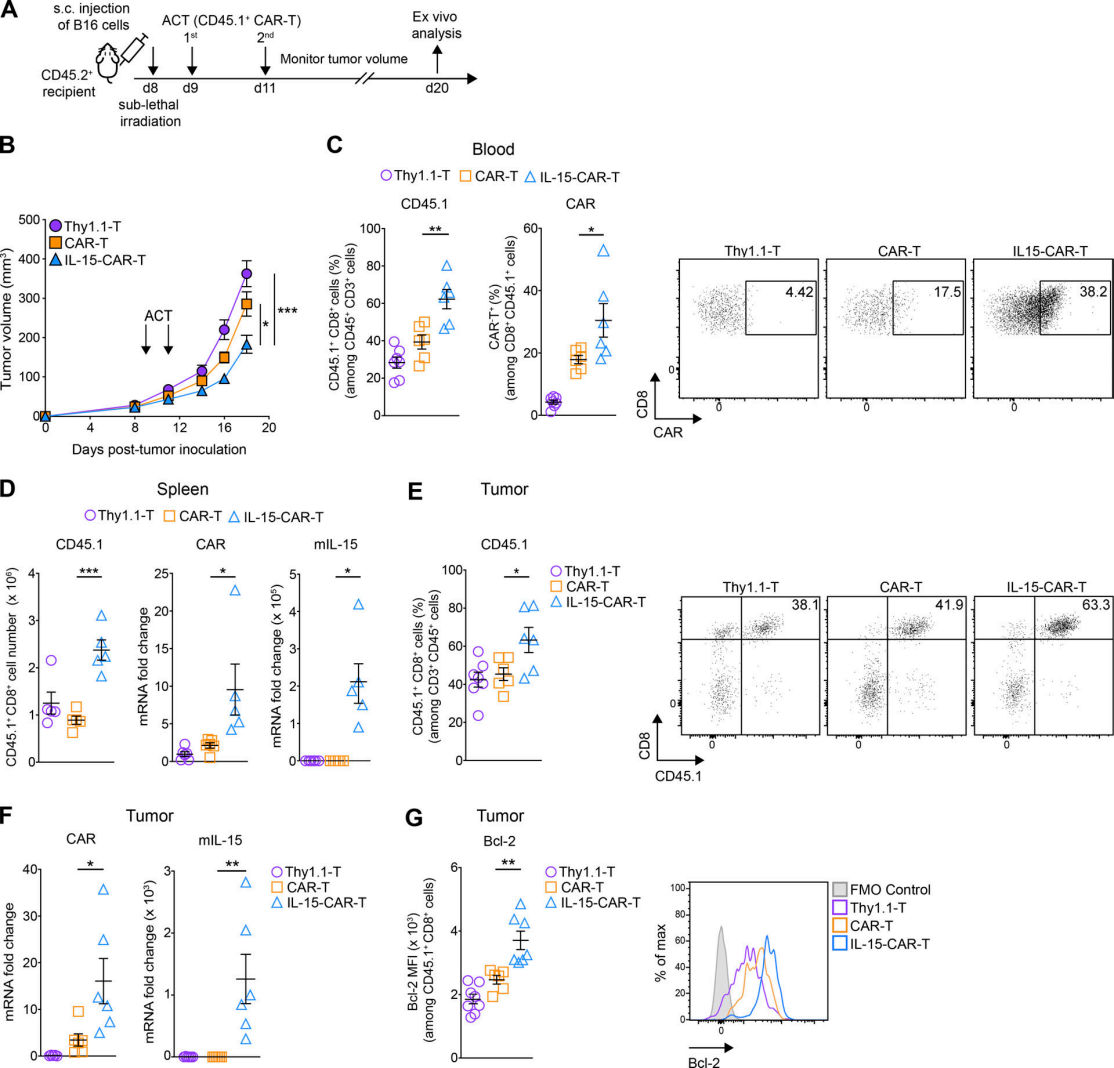
**4G-CAR-T cells achieve higher in vivo tumor control and persistence than 2G-CAR-T cells. (A)** Schematic of ACT study and ex vivo analysis. **(B)** Assessment of tumor control over time for the different treatment groups. Results are expressed as mean tumor volume (mm^3^ ± SEM) with *n* = 18 mice per group pooled from three independent experiments. The experiment was repeated four times**. (C)** Abundance of transferred T cells in the blood (day 11 after transfer), as measured by CD45.1 staining (left) and CAR expression levels (right) (*n* = 6 mice per group). Shown are representative dot plots for the assessment of CAR expression. The experiments in C (left and right) were repeated three and two times, respectively. **(D)** Number of transferred T cells (left) and relative mRNA quantification of the CAR (middle) and mIL-15 (right) transgenes in the spleens (*n* = 5–6 mice per group). The experiment in the left panel was repeated three times, and the experiments in the middle and right panels were repeated twice. **(E)** Abundance of transferred T cells in the tumors as measured by CD45.1 staining (*n* = 6–7 mice per group). Shown are representative dot plots for the frequency of tumor-residing CD45.1^+^ CD8^+^ T cells. The experiment was repeated three times. **(F)** Relative mRNA quantification of the CAR (left) and mIL-15 (right) transgenes in the tumors (*n* = 5–6 mice per group). The experiments were repeated twice. **(G)** Expression levels of Bcl-2 (MFI) in tumor-infiltrating transferred T cells (*n* = 6–8 mice per group) along with representative histograms. The experiment was repeated three times. All experimental data show mean values ± SEM. Statistical analyses were performed using a two-way repeated measures ANOVA with Tukey post hoc correction test (B) and a one-way ANOVA with Tukey post hoc correction test (C–G): *, P < 0.05; **, P < 0.01; ***, P < 0.001.

We further tested the 4G- and 2G-CAR-T cells in vivo against subcutaneous B16 melanoma tumors. Briefly, on day 8 after tumor cell injection, with tumors approaching 20–40 mm^3^ in volume, CD45.2^+^ C57BL/6 mice were lymphodepleted by sublethal total body irradiation and subsequently received two intravenous T cell injections (8–9 × 10^6^ CD45.1^+^ cells at each injection; Fig. 7 A). In mice treated with control T cells, the tumors grew rapidly, while modest tumor control was observed in mice that received 2G-CAR-T cells, similar to previous reports for this tumor vasculature targeting CAR (Chinnasamy et al., 2010, 2012). Mice treated with 4G-CAR-T cells, however, had significantly attenuated tumor growth (Fig. 7 B).

**Figure S4.**
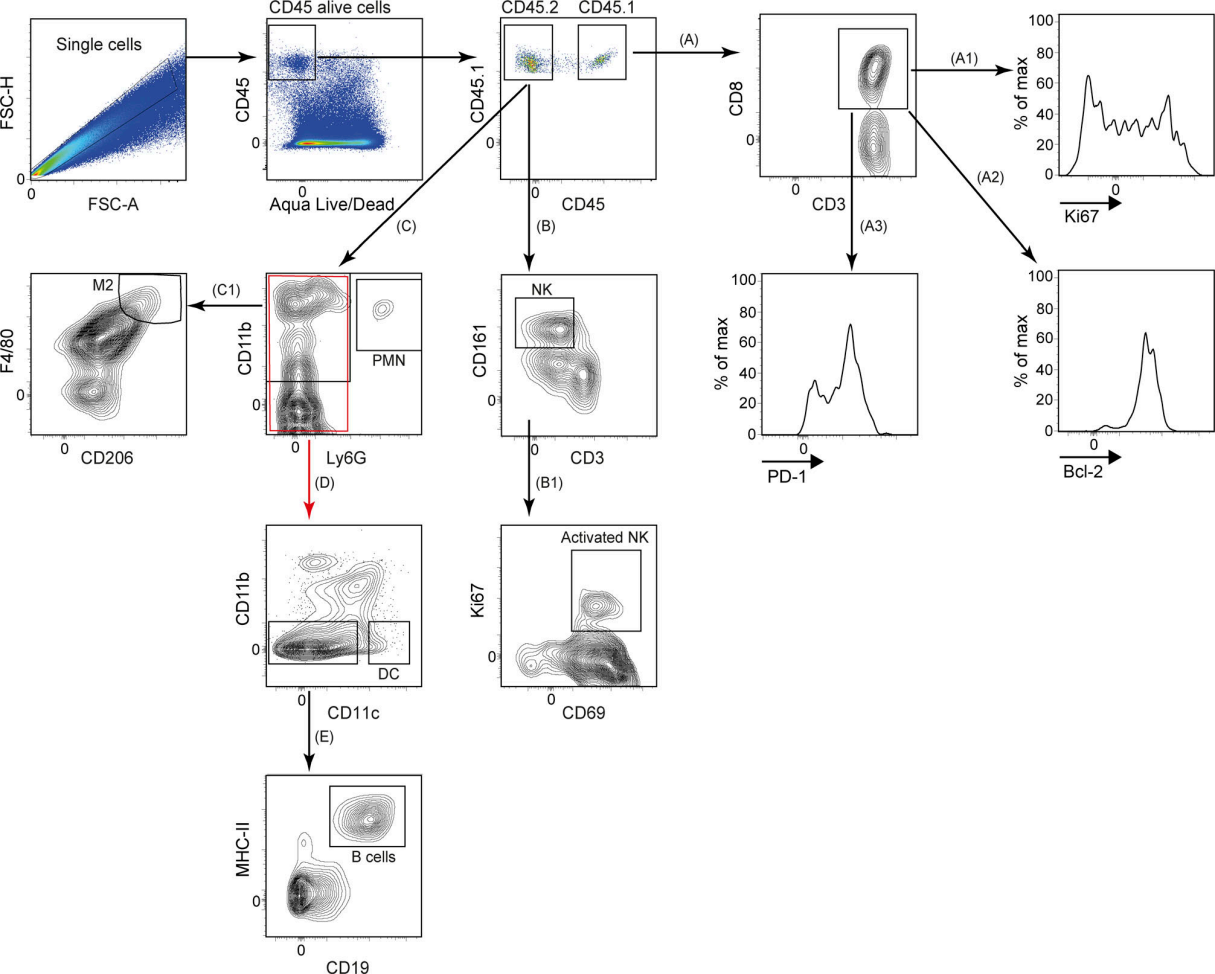
**Workflow demonstrating the ex vivo gating strategy.** Shown is the flow cytometry gating strategy for evaluating the phenotype of adoptively transferred CD45.1^+^ CD8^+^ T cells (A1–A3), the activation status of NK cells (B1) and the identification of M2 macrophages (C1), DCs (D), and B cells (E). FSC-A, forward scatter area; FSC-H, forward scatter height.

Ex vivo analysis of transferred CD45.1^+^ T cells in the blood, spleen, and tumor on day 11 after ACT revealed significantly higher engraftment of 4G- than 2G-CAR-T cells and control T cells (Fig. 7, C–E). In addition, CAR expression levels were higher for 4G- than 2G-CAR-T cells in blood, spleen, and tumor (Fig. 7, C, D, and F). Notably, we observed sustained presence of the mIL-15 transgene in the spleens and tumors of mice treated with 4G-CAR-T cells (Fig. 7, D and F). Finally, in agreement with our in vitro data, 4G-CAR-T cells expressed significantly higher levels of the antiapoptotic protein Bcl-2 in vivo (Fig. 7 G; flow cytometry gating strategy shown in Fig. S4). Thus, mIL-15 coexpression by CAR-T cells enhances not only expansion and in vitro effector functions but also in vivo persistence and tumor control.

### IL-15–CAR-T cells are characterized by lower PD-1 expression than 2G-CAR-T cells in vivo, and they reprogram the TME

**Figure 8.**
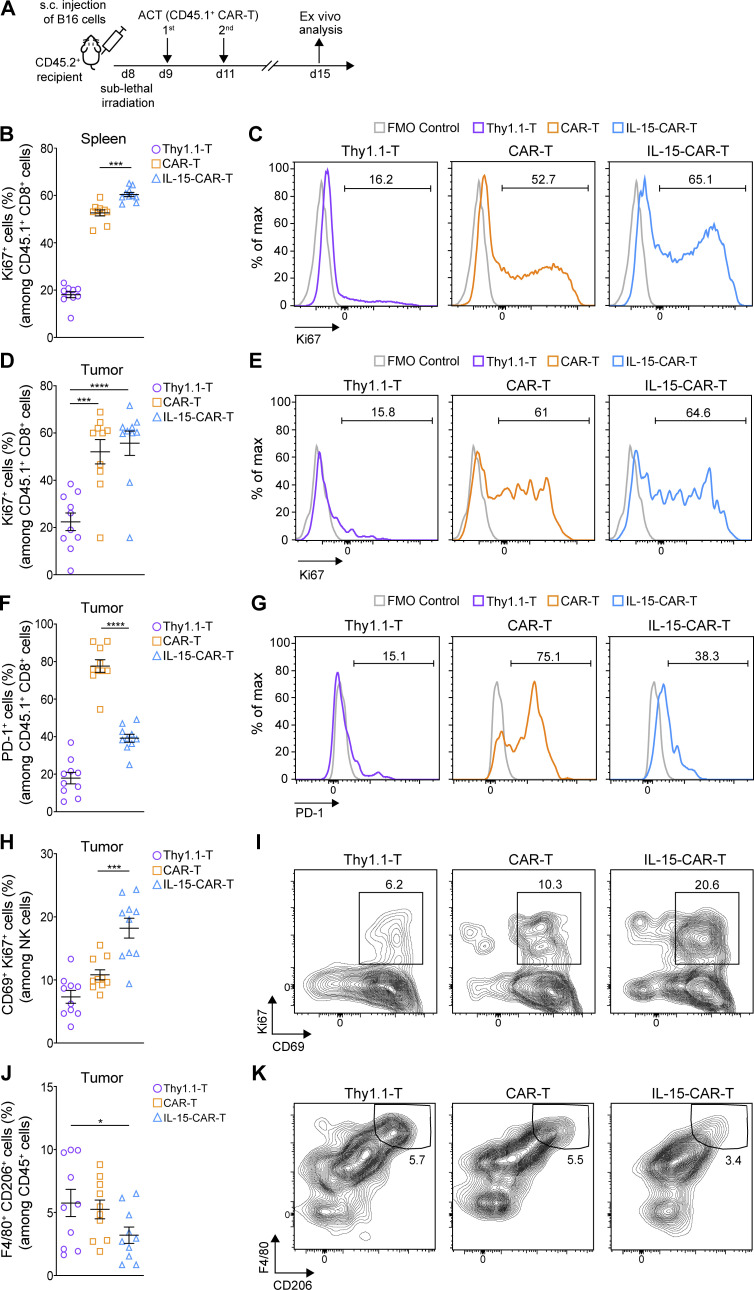
**4G-CAR-T cells coexpressing mIL-15 are characterized by lower PD-1 expression than 2G-CAR-T cells in vivo and promote TME remodeling.** **(A)** Schematic of ACT study and ex vivo analysis. **(B–E)** Assessment of Ki67 expression in the transferred T cells in the spleens (B) and tumors (D) of all treatment groups. Representative flow cytometry histograms showing the frequency of Ki67 in the transferred T cells in the spleens (C) and tumors (E). **(F)** Evaluation of PD-1 expression on the transferred T cells in the tumors. **(G)** Representative histograms showing the frequency of PD-1 among the transferred tumor-infiltrating T cells. **(H)** Assessment of the frequency of activated (CD69^+^ Ki67^+^) tumor-residing NK cells. **(I)** Representative contour plots showing the frequency of activated tumor-residing NK cells. **(J)** Assessment of the frequency of M2 (F4/80^+^ CD206^+^) tumor-infiltrating macrophages among the CD45^+^ cells. **(K)** Representative contour plots showing the frequency of tumor-residing M2 macrophages in all treatment groups. Graphs in B, D, F, H, and J present mean frequencies ± SEM of *n* = 10 mice pooled from two independent experiments. Statistical analyses were performed using a one-way ANOVA with Tukey post hoc correction test (B, D, F, and H) and a one-way ANOVA followed by Fisher’s least significant difference test (J): *, P < 0.05; **, P < 0.01; ***, P < 0.001; ****, P < 0.0001. FMO, fluorescence minus one.

**Figure S5.**
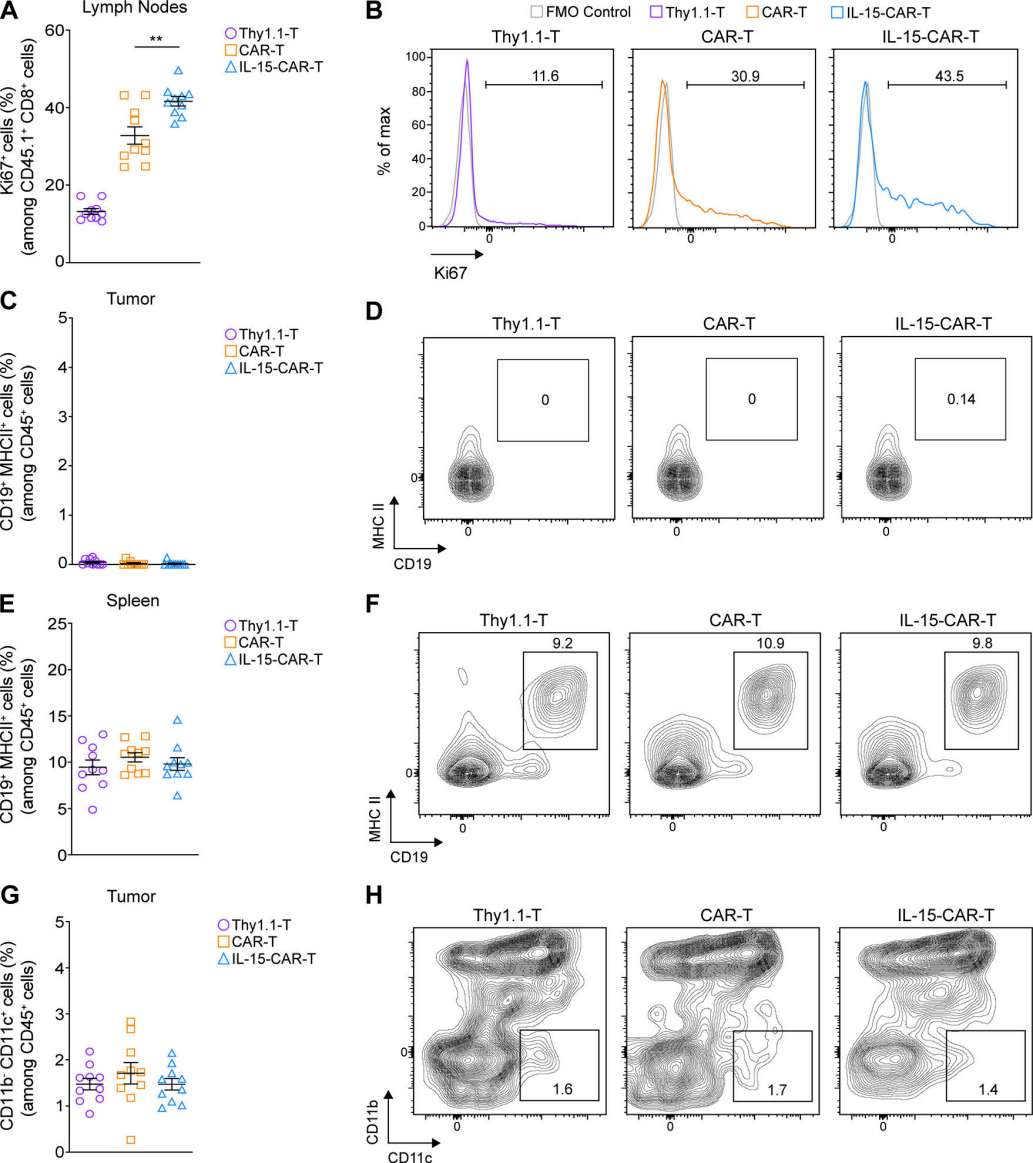
**Ex vivo analysis of CAR-T cell–treated mice. (A and B)** Assessment of Ki67 expression in the transferred T cells in the tumor-draining lymph nodes and representative flow cytometry histograms. **(C–F)** Assessment of the frequency of B cells (CD19^+^ MHCII^+^) among CD45^+^ cells in the tumors (C) and spleens (E) of treated mice along with representative contour plots shown in D and F, respectively. **(G and H)** Assessment of the frequency of tumor-residing DCs (CD11b^−^ CD11c^+^) among CD45^+^ cells of treated mice (G) and representative contour plots (H). Graphs in A, C, E, and G present mean frequencies ± SEM obtained at day 6 after ACT from *n* = 10 mice pooled from two independent experiments. Statistical analysis in A was performed using a one-way ANOVA with Tukey post hoc correction test: **, P < 0.01.

Finally, we sought to comprehensively evaluate the effect of mIL-15 coexpression on CAR-T cells in vivo and to determine if endogenous immune cells are also impacted. Following the same ACT strategy as demonstrated above (Fig. 8 A), we observed that 4G-CAR-T cells in the spleen (Fig. 8, B and C) and tumor-draining lymph nodes (Fig. S5, A and B) exhibited a higher frequency of Ki67 (cellular marker for proliferation) than 2G-CAR-T cells. In the tumor, despite that Ki67 expression levels were similar for both 4G- and 2G-CAR-T cells (Fig. 8, D and E), the 4G-CAR-T cells displayed significantly lower levels of PD-1 (Fig. 8, F and G). Analysis of endogenous immune infiltrate revealed significantly higher coexpression of CD69 and Ki67 by natural killer (NK) cells in 4G- as compared with 2G-CAR-T cell–treated tumors (Fig. 8, H and I). In addition, in 4G-CAR-T cell–treated mice there were lower levels of tumor-residing M2 (F4/80^+^ CD206^+^) macrophages, which are often associated with immunosuppression in the TME (Fig. 8 J, K). Both the activation of NK cells and lower levels of M2 macrophages may contribute to tumor control in the context of 4G-CAR-T cell transfer. Tumor-residing B cells (CD19^+^ MHC II^+^) were not detected (Fig. S5, C and D), and there were no differences in splenic B cell frequency in any of the treated mice (Fig. S5, E and F). Finally, similar frequencies of tumor-residing dendritic cells (DCs; CD11b^−^ CD11c^+^) were observed among the control and CAR-T cell–treated mice (Fig. S5, G and H). The flow cytometry gating strategy for the ex vivo characterization of the different immune cell populations is shown in Fig. S4. Thus, 4G-CAR-T cells coexpressing mIL-15, in addition to conferring enhanced tumor control as compared with 2G-CAR-T cells, also reprogram the TME in favor of protective endogenous immunity.

## Discussion

CAR-T cell therapy has yielded unprecedented clinical responses against some hematological malignancies, but not against epithelial-derived solid tumors (Irving et al., 2017). Rational combinatorial treatments and innovative CAR-T cell coengineering strategies (Lanitis et al., 2020) offer solutions for overcoming obstacles in the solid TME, but these are best evaluated in immunocompetent mice to enable the interplay of the endogenous immune system. In this study, we have presented optimized conditions for murine T cell activation, retroviral transduction, and expansion that allowed us to achieve consistently high and stable transgene expression levels, as well as robust expansion of both 2G- and 4G-CAR-T cells having a predominantly T_CM_ cell phenotype, which is favored for ACT (Melchionda et al., 2005; Gattinoni et al., 2005; Zhou et al., 2005). We have also elucidated the beneficial impact of mIL-15 coexpression by murine CAR-T cells both in vitro and in vivo.

Retroviral vectors, most commonly derived from the murine stem cell virus (MSCV), a derivative of the Moloney murine leukemia virus, have proven to be the most effective approach for stably introducing genes into murine T cells (Kerkar et al., 2011). Lentivirus, however, has demonstrated poor gene transfer in murine T cells, likely due to impaired completion of reverse transcription and of nuclear import of the viral preintegration complex (Baumann et al., 2004; Tsurutani et al., 2007). Most examples of efficient murine T cell retroviral transduction are for small and easily expressed reporter genes like GFP (Kurachi et al., 2017; Zhang et al., 2003) or 1G-CARs comprising the CD3ζ endodomain only (Lee et al., 2009). Retrovirus-mediated expression of 2G-CARs has proven less robust both in terms of percentage transduction and expression level per T cell (Kochenderfer et al., 2010; Davila et al., 2013; Fu et al., 2013). Moreover, the long-term stability of CAR expression by murine T cells has not previously been thoroughly evaluated (Kusabuka et al., 2016; Kochenderfer et al., 2010).

Despite that it is common procedure to concentrate lentivirus via ultracentrifugation, this is usually not performed for CAR-encoding retroviruses. In this study, we demonstrated that retrovirus can be efficiently concentrated, leading to significantly improved CAR transduction efficiencies. We further observed a correlation between CD8^+^ T cell activation levels (the highest level was achieved by αCD3/CD28 bead stimulation) and transduction efficiency. Previous studies have presented CAR expression early after transduction (2–3 d; Tran et al., 2013; Kusabuka et al., 2016; Kochenderfer et al., 2010) and thus cannot distinguish from pseudo-transduction (Case et al., 1999; Costello et al., 2000). In addition, some studies have applied antibiotic selection for enrichment of CAR-T cells (Kusabuka et al., 2016) or have measured GFP (or other markers) that can overestimate transduction efficiency. Here, we have demonstrated robust, long-term CAR expression in murine T cells by staining with recombinant target antigen and in the absence of any selection/enrichment method.

In this study, we have also shown the utility of the common γ-chain cytokines hIL-7/IL-15 for enhanced CAR-T cell expansion and survival, as well as for promoting a T_CM_ cell phenotype and ameliorating effector function. Others have reported superior tumor control by IL-7/IL-15 than IL-2–expanded T cells (Cha et al., 2010; Gattinoni et al., 2005; Mueller et al., 2008). It has also been previously demonstrated that exposure of murine T cells to IL-2 can potentiate apoptosis by suppressing the inhibitor of Fas signaling, FLIP (FLICE-inhibitory protein), and enhancing the expression of the proapoptotic molecule Fas ligand (Lenardo, 1991; Refaeli et al., 1998). In contrast, IL-7 and IL-15 inhibit activation-induced cell death, support the proliferation and survival of T cells (Waldmann, 2015; Jiang et al., 2004; Cha et al., 2010), promote a T_CM_ cell phenotype characterized by longer telomeres, and elevate T cell persistence and antitumor efficacy (Melchionda et al., 2005; Gattinoni et al., 2005; Zhou et al., 2005; Klebanoff et al., 2004; Le et al., 2009). Similarly, it has been shown that IL-7 and IL-15 enable enhanced human CAR-T cell effector function upon antigen recognition (Xu et al., 2014; Zhou et al., 2019) and that exogenous IL-15 can expand anti-CD19 CAR-T cells in treated patients by up to 180-fold (Ramanayake et al., 2015). Contradictory reports of lower murine T cell function in vitro following culture in IL-7/IL-15 versus IL-2 alone are presumably due to the method of T cell stimulation used, differences in the concentration of IL-2 used, and the duration of expansion (Cha et al., 2010; Gattinoni et al., 2005; Mueller et al., 2008).

We further showed that our methodologies enable the efficient coexpression of mIL-15 and a CAR (encoded by a bicistronic vector) in murine T cells. Human CAR-T cells coexpressing hIL-15 as a fusion protein tethered to the cell surface, or in a secreted form, have previously demonstrated enhanced expansion and persistence upon antigen stimulation (both in vitro and in vivo), as well as increased tumor control (Hoyos et al., 2010; Markley and Sadelain, 2010). As such, there are high expectations for clinical efficacy of IL-15–CAR-T cells. In nonactivated murine 4G-CAR-T cells, we observed very low levels of mIL-15 in the culture supernatant, but upon antigenic stimulation, significantly higher amounts were detected, in line with reports for hIL-15 CAR-T cells (Krenciute et al., 2017; Hoyos et al., 2010). Elevated levels of pSTAT5 in the 4G- versus 2G-CAR-T cells indicated active signaling by cytokine/receptor engagement. The functional integrity of the coexpressed mIL-15 was further supported by enhanced 4G-CAR-T cell proliferation and survival, possibly due to up-regulation of the antiapoptotic molecule Bcl-2 (Wu et al., 2002; Shenoy et al., 2014). In addition, mIL-15 coexpression promoted a T_CM_ cell phenotype, limited PD-1 up-regulation, and conferred superior effector function upon antigenic challenge.

The culture methods presented herein comprising hIL-7/hIL-15 in the medium permitted efficient murine CAR-T cell expansion, which was significantly reinforced upon mIL-15 coexpression by CAR-T cells. This enabled us to further investigate the efficacy of 4G-CAR-T cells in vivo against B16 melanoma tumors. We observed higher tumor control and persistence of 4G- as compared with the 2G-CAR-T cells and sustained expression of the mIL-15 transgene. Moreover 4G-CAR-T cells exhibited higher Bcl-2 levels, in line with our in vitro data, suggesting that mIL-15 can render CAR-T cells more resistant to apoptosis in vivo*.* The coexpression of mIL-15 was also associated with significantly lower up-regulation of PD-1, an inhibitory receptor that can impair T cell function in the TME (Ahmadzadeh et al., 2009).

Finally, evaluation of endogenous tumor immune infiltrate revealed a significantly higher frequency of activated (CD69^+^ Ki67^+^) NK cells and fewer M2 (F4/80^+^ CD206^+^) macrophages upon 4G- versus 2G-CAR-T cell transfer. As NK cells are associated with delayed melanoma tumor growth (Nath et al., 2019), and M2 macrophages have been shown to contribute to tumor progression and metastasis (Poh and Ernst, 2018), the observed TME remodeling upon 4G-CAR-T cell transfer is favorable for tumor control. Our findings are consistent with prior studies. For example, coadministration of IL-15 with tumor-directed monoclonal antibodies enhanced Ab-dependent cellular cytotoxicity by augmenting both NK cell and macrophage activation (Zhang et al., 2018). In another study, it was shown that the absence of IL-15 in immunocompetent mice promotes the formation of M2 macrophages (Gillgrass et al., 2014).

In summary, we have presented comprehensive and highly reproducible methods for efficient retroviral transduction and robust expansion of murine CAR-T cells endowed with favorable properties for ACT studies in immunocompetent mice. We further demonstrated that coexpression of mIL-15 directly promotes CAR-T cell fitness and function and remodels the TME to favor tumor control. As it is becoming apparent that endogenous immunity can play a critical role in either suppressing or supporting CAR-T cell function in the TME (Kuhn et al., 2019), comprehensive studies in immunocompetent mice are critical for accelerating the translation of effective CAR therapies to the clinic.

## Materials and methods

### Cell culture

The murine brain endothelioma cell line bEnd3, the murine immortalized heart endothelial cell line H5V, and the murine leukemia cell line C1498 were cultured in DMEM-GlutaMAX comprising 4,500 mg/liter glucose and 110 mg/liter sodium pyruvate and supplemented with 10% heat-inactivated FBS (Gibco, Thermo Fisher Scientific), 100 U/ml penicillin, and 100 µg/ml streptomycin sulfate. The melanoma cell line B16-F10 was grown as a monolayer in DMEM-GlutaMAX supplemented with 10% FBS, 100 U/ml of penicillin, and 100 µg/ml streptomycin sulfate. Cells were passaged twice weekly to maintain them under exponential growth conditions and were routinely tested for mycoplasma contamination. The Phoenix Eco retroviral ecotropic packaging cell line, derived from immortalized normal human embryonic kidney cells, was maintained in RPMI 1640-Glutamax medium supplemented with 10% FBS, 100 U/ml penicillin, and 100 µg/ml streptomycin sulfate. Primary murine T cells were cultured in RPMI 1640-Glutamax medium supplemented with 10% FBS, 100 U/ml penicillin, 100 µg/ml streptomycin sulfate, 1 mM sodium pyruvate, 50 µM β-mercaptoethanol, and 10 mM nonessential amino acids (referred to as murine T cell culture medium). Murine T cell culture medium was further supplemented with human cytokines as described in the method for T cell expansion.

### CAR construction

The retroviral vector pMSGV (murine stem cell virus [MSCV]–based splice-gag vector) comprising the MSCV LTR was used as the backbone for all CAR constructs. A 2G-CAR consisting of the anti-VEGFR-2 scFv, DC101, the CD8α hinge (H), and TM region, followed by the EDs of CD28 and CD3ζ (DC101-28-z), was kindly provided by Dr. Steven A. Rosenberg (National Cancer Institute, Bethesda, MD; Chinnasamy et al., 2010). The DC101-28-z CAR was built by PCR amplification of a 362-bp fragment from the 2G construct with the primers: 5′-ACG​CGC​GGC​CGC​AAC​TAC​TAC​CAA​GC-3′ and 5′-ACG​CGT​CGA​CGG​GGC​GGT​ACG​CTG​CAA​AGT​CTC-3′ followed by NotI and SalI digestion of both the PCR product and the parental 2G vector, gel purification, and ligation. To generate the 4G-CAR construct encoding both mIL-15 and the VEGFR-2–directed CAR (mIL-15-T2A-DC101-28-z), a gene-string encoding the murine Igκ leader sequence followed by codon-optimized mIL-15 and T2A, flanked by XhoI and EcoRI restriction sites at the 5′ and 3′ ends, respectively, was synthesized. The DC101-28-z construct and fragment were then digested (XhoI and EcoRI), gel purified, and ligated together. All genes strings were synthesized by GeneArt AG, and all constructs were fully sequenced by Microsynth AG.

### Retrovirus production

High-titer, replication-defective retrovirus was produced and concentrated as depicted in Fig. 1. Briefly, Phoenix Eco cells were seeded at 10^7^ per T-150 tissue culture flask in 35 ml culture medium (Fig. 1 A, 1) 24 h before transfection with 14.4 µg pCL-Eco Retrovirus Packaging Vector and 21.4 µg pMSGV transfer plasmid using Turbofect (Thermo Fisher Scientific; Fig. 1 A, 2). All plasmids were purified using HiPure Plasmid Filter Maxiprep Kit (Invitrogen, Thermo Fisher Scientific). For the transfection mixture, a 3:1 ratio of Turbofect/plasmid was prepared in 2 ml Opti-MEM and incubated for 30 min at room temperature (RT; Fig. 1 A, 2). Medium was then removed from T-150 flasks bearing 80–90% confluent Phoenix Eco cells and the transfection mixture was applied and incubated for 1 min, followed by addition of 31 ml fresh medium (Fig. 1 A, 2). The viral supernatant was discarded 20–24 h after transfection and replaced with 33 ml fresh medium (Fig. 1 A, 3). At 48 (Fig. 1 A, 4) and 72 h (Fig. 1 A, 5) after transfection, the supernatant was harvested, and viral particles were concentrated by ultracentrifugation for 2 h at 24,000 *g* at 4°C with a Beckman JS-24 rotor (Beckman Coulter) and resuspended in 0.4 ml murine T cell medium. The retrovirus was then used immediately, or aliquoted, frozen on dry ice, and stored at −80°C.

### Determination of viral titer

Titers were determined in a small-scale, single-round transduction (described below) of 10^5^ activated murine T cells added to 96 non-treated well plates (Corning Falcon) already loaded with threefold serial dilutions (up to 1 in 2,187) of concentrated retrovirus, in a final volume of 50 μl. After 7 d of expansion, CAR expression was evaluated by specific staining and flow cytometric analysis (described below). Titers (expressed as transducing units/milliliter) were calculated according to the formula: (*F*/100) × *N* × 20 × *D*, where *F* is the percentage of CAR^+^ T cells, *N* is the number of T cells at the time of transduction (usually 10^5^ cells), and *D* is the fold dilution of virus used in transduction.

### Murine T cell and leukemia cell transduction

As depicted in Fig. 1 B, murine T cells were isolated from single-cell suspensions of dissociated spleens from CD45.1^+^ congenic C57BL/6 mice bred in-house at the animal facility of the University of Lausanne (UNIL; Epalinges, Switzerland) using the EasySep Mouse T Cell Isolation Kit (StemCell Technologies; Fig. 1 B, 1.1). T cells were plated at 10^6^/ml in 24- or 48-well plates in T cell medium (described above) and stimulated with αCD3/CD28 Ab-coated beads (Invitrogen) at a bead to cell ratio of 2:1 and 50 IU/ml hIL-2 (Glaxo; Fig. 1 B, 1.1). Non–treated cell-culture grade 48- or 24-well plates (Corning Falcon) were precoated with 0.25 ml or 0.5 ml, respectively, of recombinant RetroNectin (Takara Bio) at a final concentration of 20 μg/ml, overnight (O/N) at 4°C (Fig. 1 B, 1.2). 1 d after T cell activation, the retronectin-precoated plates were washed with PBS, blocked with 2% BSA in PBS for 30 min at RT (Fig. 1 B, 2.1). Subsequently, plates were washed once, retrovirus was added at the MOI indicated in the figures, and plates were then spun at 2,000 *g* for 1.5 h at 32°C (Fig. 1 B, 2.2). The supernatants were then aspirated, and 0.5 to 10^6^ of 24 h activated T cells were transferred to each coated well (48- or 24-well plates; Fig. 1 B, 2.3). The plates were centrifuged for 10 min at 300 *g* and incubated O/N (Fig. 1 B, 2.3). In some experiments the transduction procedure was performed at 48 h, or at both 24 and 48 h after activation. The cultures were maintained at a cell density of 0.5 to 10^6^ cells/ml and replenished with fresh T cell medium (supplemented with hIL-2 alone or hIL-2 followed by hIL-7/IL-15 on day 2 after transduction) every other day (Fig. 1 B, 3). At day 7, CAR surface expression was assessed by flow cytometric analysis (as described below), and the rested engineered T cells were adjusted for equal expression before functional in vitro and in vivo assays (Fig. 1 B, 4).

Murine C1498 leukemia cells were transduced as described above for primary murine T cells, except that they were not activated and were maintained afterwards in DMEM-GlutaMAX complete medium at a cell density of 3 × 10^5^ viable cells/ml.

### T cell activation, expansion, viability, and phenotype assessment

Murine T cells were activated for 24 h with (i) αCD3/CD28 Ab-coated beads (Gibco, Thermo Fisher Scientific) at a bead to cell ratio of 2:1 and 50 IU/ml of hIL-2 (Glaxo), (ii) plate-coated αCD3 Ab (5 µg/ml; eBioscience) plus soluble αCD28 Ab (2 µg/ml; eBioscience) and hIL-2 (50 IU/ml), or (iii) Concanavalin A (2 µg/ml; Sigma), hIL-7 (1 ng/ml; Miltenyi) and 50 IU/ml hIL-2 before transduction. Transduced T cells were cultured at a concentration of 0.5–10^6^ cells/ml in T cell medium enriched with 50 IU/ml hIL-2 only or with 10 ng/ml of both hIL-7 and hIL-15 (Miltenyi) from day 2 after transduction onwards. T cells were typically counted every 2–3 d. T cell expansion was calculated by dividing the absolute number of expanded T cells at each time point during culture by the respective number on day 0 (T cell transduction). T cell viability was assessed by trypan blue staining, and the phenotype was assessed by cell-surface staining of CD44 and CD62L followed by flow cytometric analysis.

### Cytokine release assays

Cytokine release assays were performed in triplicate in 96-well plates by co-culturing 0.5 × 10^5^ transduced T cells with 0.5 × 10^5^ target cells per well in 200 µl T cell medium. In some experiments, CD8^+^ T cells were first sorted from transduced T cells using the EasySep Mouse CD8^+^ T Cell Isolation Kit (StemCell Technologies) before co-culturing them with target cells. After 20–24 h of co-culture, the supernatants were assayed for the presence of IFN-γ and granzyme B by commercial ELISA Kits (BioLegend and eBioscience, respectively). IL-2 was measured by Cytokine Bead Array as described by the manufacturer (BD Biosciences).

### Measurement of mIL-15

For intracellular mIL-15 protein quantification, transduced T cells were washed and placed O/N in cytokine-free medium at a concentration of 10^6^ T cells/ml. The next day, the T cells were harvested, lysed via one freeze/thaw cycle, and pelleted by centrifugation, and mIL-15 was measured in the supernatant by ELISA (Murine IL-15 Standard ABTS ELISA Development Kit; Peprotech) according to the manufacturer’s instructions. mIL-15 was similarly quantified by ELISA in the supernatants of Phoenix Eco cells at 72 h after transfection and the supernatants of transduced C1498 cells at 24 h after incubation of equal numbers of transduced cells in 48-well plates. For the measurement of mIL-15 in the supernatants of T cell cultures stimulated with recombinant protein, plate wells were coated with soluble mVEGFR-2 protein (1 µg/ml in PBS) or control BSA protein (2% in PBS) for 4 h at RT and washed with PBS, followed by addition of equal numbers of transduced T cells in the coated wells. mIL-15 was measured in the supernatants by ELISA at 24 h after T cell activation. For T cell cultures stimulated with endothelial cells, mIL-15 was evaluated in the supernatants 24 h after co-culture at a CAR^+^ T cell to target ratio of 2:1.

### T cell proliferation assay

Endothelial target cells were plated at a density of 10^4^ per well in triplicate in 96-well flat-bottom plates in 200 µl total volume. The next day, T cells were labeled with 5 µM CellTrace Violet (CTV; Molecular Probes, Life Technologies) in PBS with 1% FBS at 37°C for 20 min. The reaction was then quenched with complete medium, and the cells washed and then co-cultured with the indicated established endothelial cell monolayers at a CAR^+^ T cell to target ratio of 2:1 for 4 d. Subsequently, total cells were harvested by Accutase treatment (Thermo Fischer Scientific), washed, and stained with indicated labeled Abs. Cell division was evaluated by dilution of the dye in live CD8^+^ T cells and flow cytometric analysis.

### CAR-T cell cytotoxicity

Established endothelial monolayers (15,000 cells/well of a 96-well plate) were co-cultured with CAR-T cells and control T cells at an effector to target ratio of 3:1 for 3 d. The cells were subsequently collected by Accutase treatment, washed, and stained for Annexin V and 7-aminoactinomycin D (7-AAD) using an Apoptosis Detection Kit (BD Pharmingen) according to the manufacturer’s instructions.

### Flow cytometry: Surface and intracellular staining

For flow cytometric analysis, cells were surface stained using antibodies against CD3ε (145-2C11), CD4 (GK1.5, RM4-5), CD8α (53–6.7), CD25 (PC61), CD44 (IM7), CD45.1 (A20), CD45 (30F/11), CD62L (MEL-14), CD69 (H1-2F3), IL-15-Rα (6B4C88), PD-1 (29F.1A12), Ly-6G (1A8), CD11b (M1/70), CD11c (N418), F4/80 (BM8), CD206 (C068C2), NK-1.1 (PK136), CD19 (6D5), and MHC class II (M5/114.15.2). Abs were purchased from eBioscience and BioLegend or produced in-house from hybridomas by the flow cytometry platform. DC101-CAR expression by retrovirally transduced T cells was detected by incubation with soluble mouse VEGFR-2–hIgG-Fc fusion protein (R&D Systems) followed by staining with labeled goat anti-hIgG Fc (clone HP6017; Biolegend). Thy1.1-T cells were stained in parallel as a negative control. VEGFR-2 expression by mouse endothelial cell lines was evaluated by cell-surface staining with rat anti-VEGFR-2 Ab (clone Avas12; BioLegend) and matched isotype control (Rat IgG2a κ isotype; clone RTK2758; BioLegend). For detection of phosphorylated STAT5, cells were fixed with BD Cytofix Fixation Buffer at 4°C for 15 min and permeabilized with BD Phosflow Perm Buffer III for 30 min at 4°C. Intracellular phospho-staining was performed for 1 h at RT in the dark with Ab against phospho-STAT5 (Tyr694; D47E7 XP Rabbit mAb 4322; Cell Signaling). For intracellular staining of mIL-15 (clone AIO.3; eBioscience), Bcl-2 (clone 10C4; eBioscience), and Ki67 (clone SolA15; eBioscience), T cells were fixed and then permeabilized using the FoxP3 transcription factor staining buffer set (eBioscience) according to the manufacturer’s recommendations. For the detection of mIL-15, the cells were further washed and incubated for 30 min with anti-rat IgG2a. To discriminate dead cells, 7-AAD (BioLegend) staining was performed. Live/dead fixable Aqua Dead cell staining was used to exclude dead cells in the ex vivo analysis of immune cells derived from the spleens, tumors, and tumor-draining lymph nodes according to the manufacturer’s instructions (Molecular Probes, Life Technologies). Data were acquired with a BD flow cytometer and analyzed using FlowJo software (Tree Star).

### Mouse strains

Female C57BL/6 mice aged 6–8 wk were purchased from Envigo and housed at the animal facility at the UNIL in compliance with guidelines. All in vivo experiments were conducted in accordance and with approval from the Service of Consumer and Veterinary Affairs of the Canton of Vaud. CD45.1^+^ congenic on a C57BL/6 background were bred and housed in the UNIL animal facility.

### Adoptive CAR-T cell transfer in tumor-bearing mice

B16 tumor cells were harvested with 0.05% trypsin, washed, and resuspended in PBS for injection. 10^5^ tumor cells were injected subcutaneously in the flank of C57BL/6 mice, aged 8–12 wk. 8 d later (average tumor volume, 20–40 mm^3^), the mice received 5 Gy of sublethal total body irradiation and grouped (*n* ≥ 5 mice/group) for comparative average tumor volumes. On days 9 and 11, mice were treated with intravenous transfer of 8–9 × 10^6^ CD45.1^+^ 4G- or 2G-CAR-T cells or control Thy1.1-T cells. Mice were carefully monitored, and tumor length (*L*; greatest longitudinal measurement) and width (*W*; greatest transverse measurement) measured by caliper every 2–3 d. Tumor volumes (*V*) were calculated using the formula: *V* = (*L* × *W*^2^)/2. The mean tumor volumes for each group are plotted ± SEM. Mice were sacrificed once tumors reached 1,000 mm^3^ or, according to regulation, if they became distressed or moribund.

### Evaluation of in vivo persistence of adoptively transferred T cells

Blood, spleens, tumors and tumor-draining lymph nodes were harvested from differently treated mice. Blood was treated with a RBC lysing buffer (BD Pharm Lyse, BD Biosciences), and spleens or tumor-draining lymph nodes were gently crushed through a 40-µm cell strainer followed by RBC lysis. Tumor fragments were cut into pieces with scissors and then digested in RPMI supplemented with 200 µg/ml Liberase TL (Roche) and 5 U/ml DNase I (Sigma-Aldrich) for 1 h at 37°C on a rocker, followed by passage through a 40-µm cell strainer. To calculate the absolute number of viable CD8^+^ CD45.1^+^ T cells in the spleen cell preparations, the splenic cell count was multiplied with the frequency of CD8^+^ CD45.1^+^ T cells among total live cells.

### Quantitative PCR

Cells extracted from dissociated tumors were lysed using TRIzol reagent (Invitrogen, Thermo Fisher Scientific). Total RNA was isolated using the RNeasy Mini Kit (Qiagen). After treatment with RNase-free DNase I (Qiagen), 400 ng of total RNA was reverse transcribed using PrimeScript First Strand cDNA Synthesis Kit (Takara Bio), as indicated by the manufacturer. Quantitative real-time PCR was performed according to the commercial protocol using SYBR Green Fast PCR Master Mix (Thermo Fisher Scientific) and the 7500 Fast Real-Time PCR System (Applied Biosystems). Primers to specifically amplify regions of the DC101 scFv of the CAR cassette, or the mIL-15 transgene, were designed using the GenScript website and are as follows: DC101 forward, 5′-GCA​ACC​CAA​ACT​CCT​CAT​CT-3′; DC101 reverse, 5′-TAT​CAT​CAG​CCT​CCA​CAG​GA-3′; IL-15 forward, 5′-CCA​GGA​TCT​ACA​GGC​GAC​AA-3′; IL-15 reverse, 5′-ATG​CTC​TGG​ATC​AGG​CTC​TC-3′.

PCR amplification of the housekeeping gene GAPDH was performed as a control, and to allow normalization of samples. The following primers were used for GAPDH: GAPDH forward, 5′-AGG​TCG​GTG​TGA​ACG​GAT​TTG-3′; GAPDH reverse, 5′-TGT​AGA​CCA​TGT​AGT​TGA​GGT​CA-3′. Each sample was run in triplicate, and each experiment included three nontemplate control wells. The relative mRNA levels (fold change) of each transgene among the different samples were quantified using the comparative 2^−ΔΔCt^ method.

### Statistical analysis

Statistical analyses were performed using GraphPad Prism 8 software. Analysis of differences between two groups was performed using an unpaired two-tailed Student’s *t* test. Statistical analyses of three or more groups were performed using a one-way ANOVA followed by the post hoc test stated in the figure legends. Statistical analysis of tumor growth curves was performed using a two-way repeated-measures ANOVA with Tukey post hoc correction test. Significance levels are indicated in the figures (*, P < 0.05; **, P < 0.01; ***, P < 0.001; ****, P < 0.0001).

### Online supplemental material

Fig. S1 shows the impact of different activation methods on the expression of Ki67 and CAR by transduced T cells. Fig. S2 shows the comparison of effector functions between hIL-7/IL-15– and hIL-2–expanded CD8^+^ CAR-T cells. Fig. S3 shows the absence of IL-15-Rα expression on murine C1498 leukemia cells, the expression of Bcl-2 by 4G- and 2G-CAR-T cells, and the impact of increasing concentrations of hIL-15 in the medium on the expansion of 2G- and 4G-CAR-T cells. Fig. S4 shows the flow cytometry gating strategy for the ex vivo analysis of the different immune cell populations. Fig. S5 shows the frequency of Ki67 expression in 2G- and 4G-CAR-T cells as well as control Thy1.1 T cells in the tumor-draining lymph nodes extracted from all the treatment groups.
